# Supplementary material for: Glutamylation of centrosomes ensures their function by recruiting microtubule nucleation factors
Source: EMBO J. 2025 Apr 14;44(10):2976–96. doi: 10.1038/s44318-025-00435-y (PMC12084555; doi:10.1038/s44318-025-00435-y)
Supplement: Supplementary file 1 — Table EV1 [file 44318_2025_435_MOESM1_ESM.docx]

Table EV1. ON-TARGETplus siRNAs used in this study

| siRNA | Cat. # | Targeting mRNA sequence 5'->3' |
| --- | --- | --- |
| mTTLL5 ON-TARGETplus siRNA | J-3669-05 | GGACAACAAUAUUCGAGUA |
|  | J-3669-07 | GGAGGAGAAUGAUCGGAGA |
| ON-TARGETplus Non-targeting siRNA pool | D-001801-10-20 | UGGUUUACAUGUCGACUAA  UGGUUUACAUGUUGUGUGA  UGGUUUACAUGUUUUCUGA  UGGUUUACAUGUUUUCCUA |
