# Supplementary material for: Glutamylation of centrosomes ensures their function by recruiting microtubule nucleation factors
Source: EMBO J. 2025 Apr 14;44(10):2976–96. doi: 10.1038/s44318-025-00435-y (PMC12084555; doi:10.1038/s44318-025-00435-y)
Supplement: Supplementary file 2 — Table EV2 [file 44318_2025_435_MOESM2_ESM.docx]

Table EV2. Primers for qPCR analysis

| cDNA of interest | Primer | Primer sequence 5'->3' | Product size (bp) |
| --- | --- | --- | --- |
| mGAPDH | mGAPDH-Q520F | GCATCCTGCACCACCAACTGCTTAGC | 115 |
|  | mGAPDH-Q634R | CAGTCTTCTGGGTGGCAGTGATGGC |  |
| mTTLL5 | mTTLL5-Q3159F | GAAGCAGGCAGCCAGGCAGTATTC | 154 |
|  | mTTLL5-Q3312R | GTTGGGCCACTAGGACTGATGACAG |  |
