# Supplementary material for: Glutamylation of centrosomes ensures their function by recruiting microtubule nucleation factors
Source: EMBO J. 2025 Apr 14;44(10):2976–96. doi: 10.1038/s44318-025-00435-y (PMC12084555; doi:10.1038/s44318-025-00435-y)
Supplement: Supplementary file 3 — Appendix [file 44318_2025_435_MOESM3_ESM.pdf]

Appendix to

Centrosomal Glutamylation Recruits the Microtubule Nucleation Factors to Ensure Its Functions

Shi-Rong Hong, Yi-Chien Chuang, Wen-Ting Yang, Chiou-Shian Song, Hung-Wei Yeh, Bing-Huan Wu, I-Hsuan Lin, Po-Chun Chou, Shiau-Chi Chen, Lohitaksh Sharma, Ruei-Zhen Lu, Rou-Ying Li, Ya-Chu Chang, Kuan-Ju Liao, Hui-Chun Cheng, Wong-Jing Wang, Lily Hui-Ching Wang, Yu-Chun Lin

Table of content:

Appendix Figure S1 2
Appendix Figure S2 3
Appendix Figure S3 5
Appendix Figure S4 7
Appendix Figure S5 8
Appendix Figure S6 9
Appendix Figure S7 11
Appendix Figure S8 12
Appendix Figure S9 13
Appendix Figure S10 14
Appendix Figure S11 16
Appendix Figure S12 17
Key resources table 18

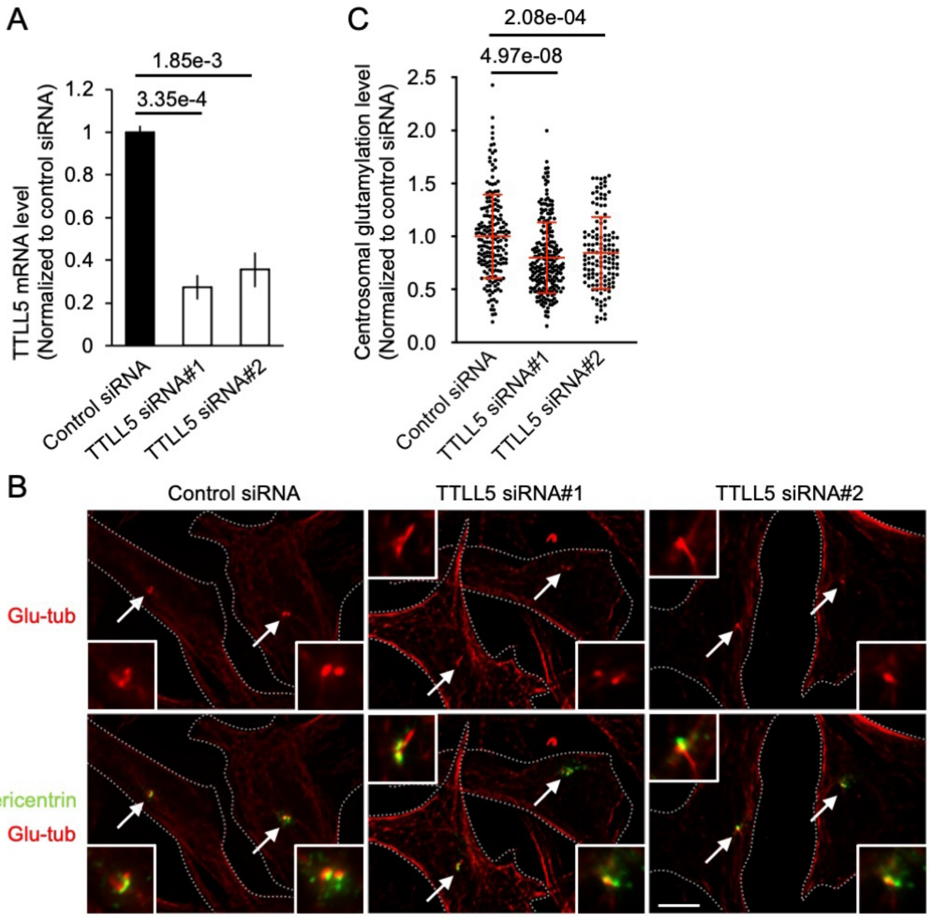

Appendix Figure S1. Depletion of TTLL5 slightly reduces glutamylation at centrosomes

A. NIH3T3 cells were transfected with control siRNA or two TTLL5 siRNAs. After 72 h of transfection, TTLL5 mRNA level was measured by quantitative PCR. Data represent the mean ± S.E.M. n = 9, 8, and 9 dishes from left to right; Three to four independent experiments.

B. Maximum intensity projection of glutamylated tubulin immunofluorescence (Glu-tub; red) and Pericentrin (marker of centrosomes; green) in NIH3T3 cells transfected with the indicated siRNAs. Arrows: centrosome regions. Insets show higher-magnification images of the centrosomes/basal-body regions. Dashed lines indicate the cell boundary. Scale bar, 10 μm.

C. Quantification of centrosomal glutamylation relative to control siRNA–transfected cells. Data (red) represent the mean ± S.E.M. n = 188, 228, and 137 cells from left to right. Three independent experiments.

Student's *t*-tests were performed, and *P* values are indicated.

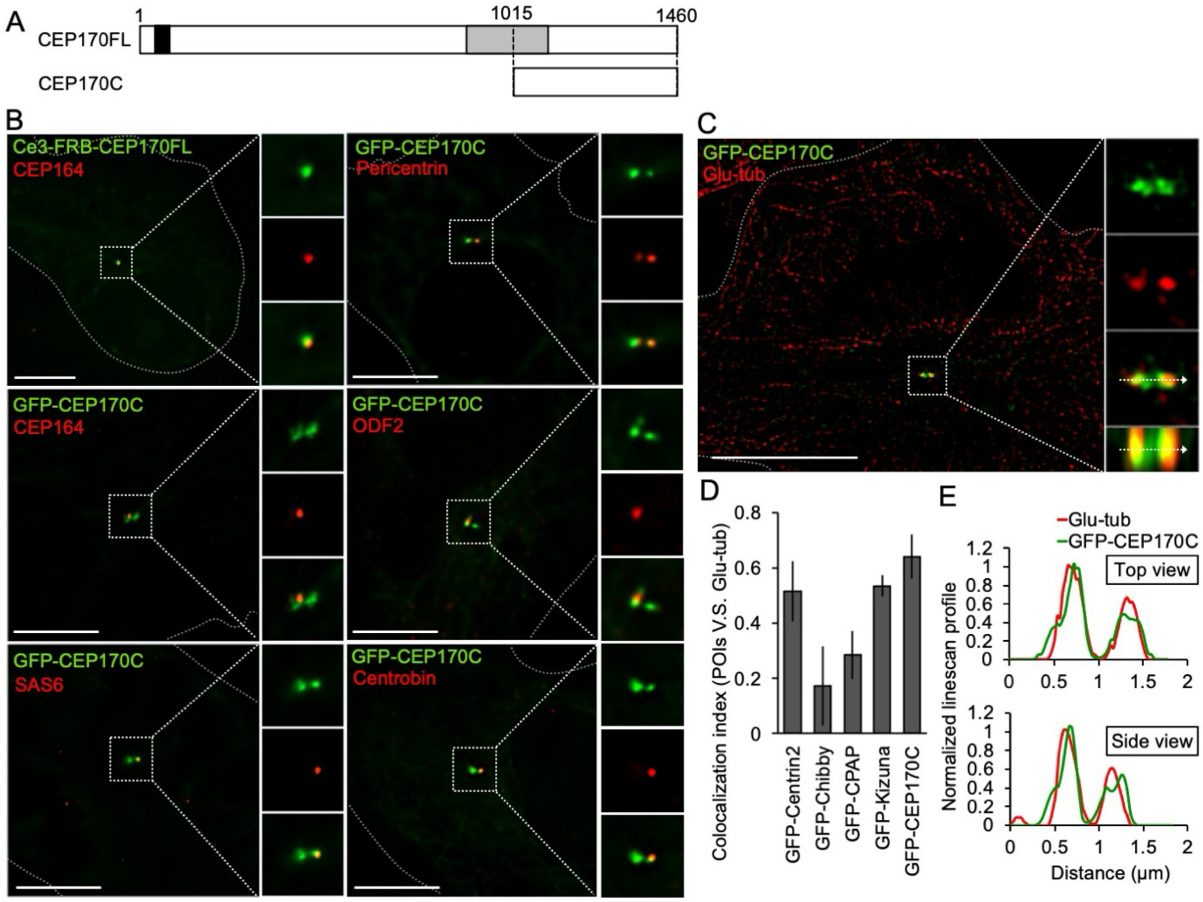

Appendix Figure S2. CEP170C localizes to glutamylated sites of centrosomes

A. Diagram of full-length CEP170 (CEP170FL; residues 1–1460) and its C-terminal portion (CEP170C; residues 1015–1460). The black and gray boxes represent the forkhead-associated domain and serine-rich domain, respectively.

B. NIH3T3 cells transfected with GFP-CEP170FL or GFP-CEP170C (green) were immunostained with antibody against CEP164, Pericentrin, ODF2, SAS6, and Centrobin (red), respectively. The right panels show magnified images of the areas outlined by the dashed squares. Scale bar, 10 μm.

C. 3D-SIM image of NIH3T3 cells transfected with GFP-CEP170C (green) and immunostained with antibody GT335 (red). The right panels show magnified images of the areas outlined by the dashed squares. Scale bar, 10 μm.

D. Colocalization index between glutamylated tubulin and the indicated GFP-tagged proteins. Data represent the mean ± S.E.M. n = 5, 3, 3, 4, and 4 cells from left to right. Two independent experiments.

E. Normalized Linescan intensity profiles of GFP-CEP170C (green) and glutamylated tubulin (red) along the dashed line in (C).

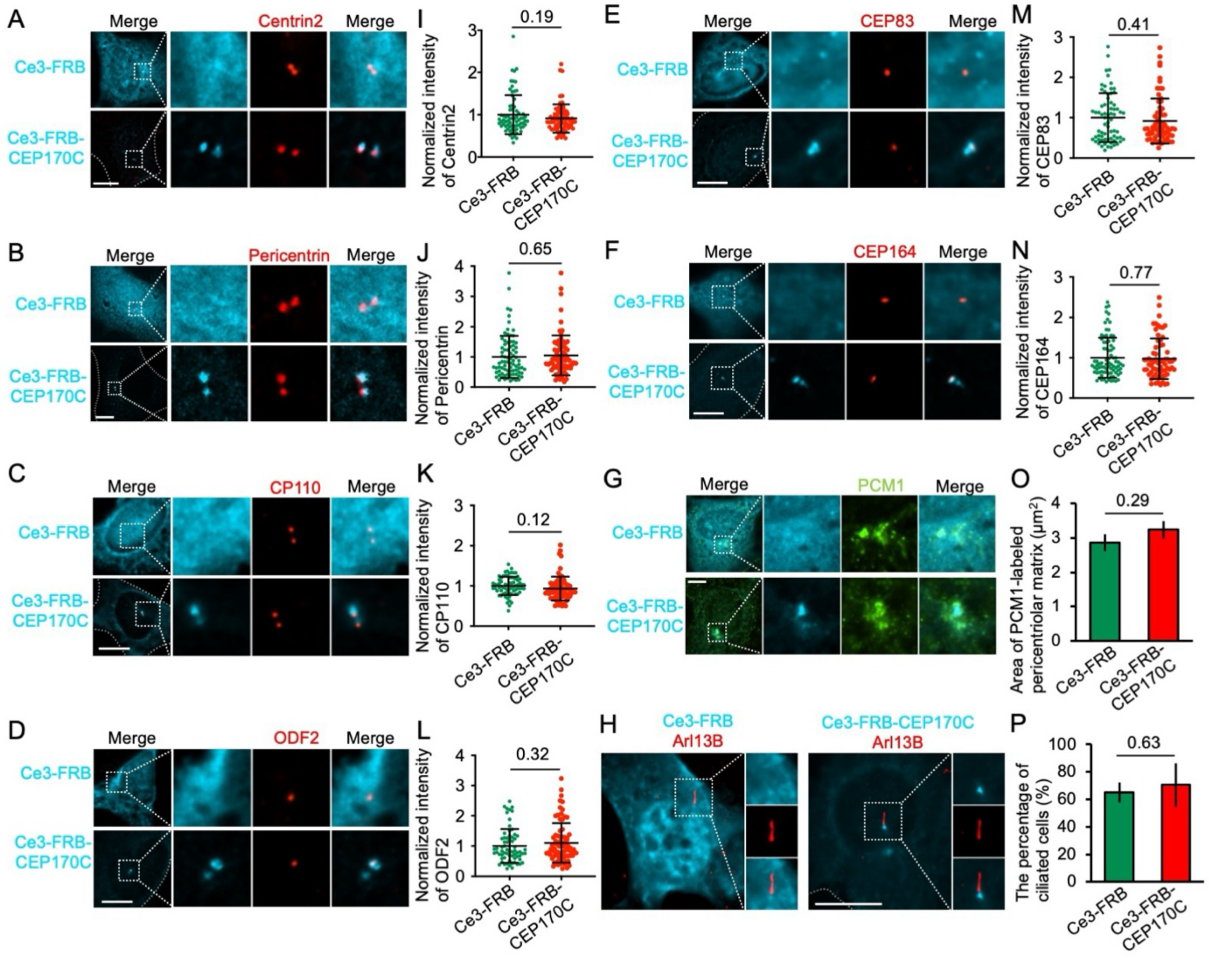

Appendix Figure S3. Expression of Cerulean3-FRB-CEP170C does not induce adverse effects on centrosomes and cilia

A-H. NIH3T3 cells were transfected with Ce3-FRB or Ce3-FRB-CEP170C. Transfected cells at 80–90% confluency were immunostained with antibodies against Centrin2 (A), Pericentrin (B), CP110 (C), ODF2 (D), CEP83 (E), CEP164 (F), and PCM1 (G), respectively. Transfected cells were serum-starved for 24 h followed by immunofluorescence staining with anti-Arl13B (H). The right panels show magnified images of the areas outlined by the dashed squares. Scale bar, 10 μm.

I-N. Normalized intensity of the indicated protein in transfected cells. Data represent the mean ± S.D. n= 142 (I), 159 (J), 146 (K), 147 (L), 138 (M), and 149 cells (N).

O. Area of PCM1-positive centriolar satellites after transfection with Ce3-FRB or Ce3-FRB-CEP170C, respectively. Data represent the mean ± S.E.M. n = 37 and 34 cells in the Ce3-FRB and the Ce3-FRB-CEP170C groups, respectively. Three independent experiments.

P. Length of primary cilia in cells expressing Ce3-FRB or Ce3-FRB-CEP170C, respectively. Data (black) represent the mean ± SD. n = 486, 257 cells in the Ce3-

FRB and the Ce3-FRB-CEP170C groups, respectively. Three independent experiments.

Student's *t*-tests were performed, and *P* values are indicated.

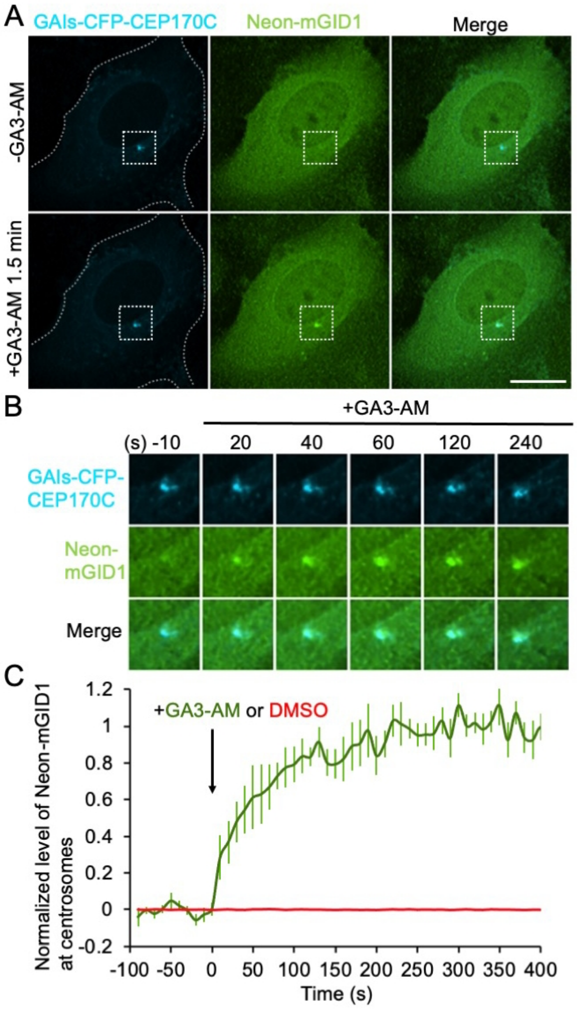

Appendix Figure S4. Translocation of cytosolic proteins to centrosomes via a rapamycin orthogonal CID system

A. NIH3T3 cells co-transfected with GAIs-CFP-CEP170C and Neon-mGID1 were treated with GA3-AM (100 μM) for 1.5 min. Dashed lines indicate the cell boundary. Scale bar, 10 μm.

B. Video frames of enlarged images of the centrosome region shown in the dashed squares in (A).

C. Normalized fluorescence intensity of Neon-mGID1 at centrosomes upon treatment with 0.1% DMSO (red) or GA3-AM (100 μM; green). Data represent the mean ± S.E.M. n = 5 cells in each of the DMSO and GA3-AM groups. Three independent experiments.

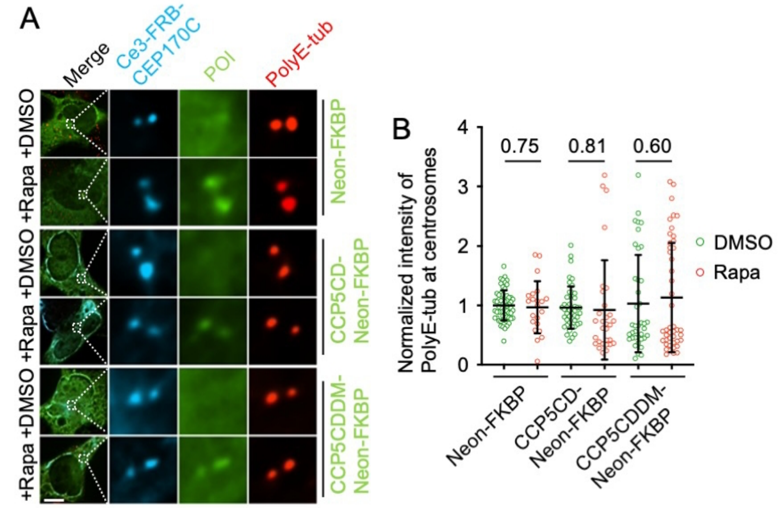

Appendix Figure S5. Recruitment of CCP5CD to centrosomes does not affect the polyglutamylated tubulin levels.

A. COS7 cells were co-transfected with Ce3-FRB-CEP170C (green) and the indicated constructs, followed by treatment with either 0.1% DMSO or 100 nM rapamycin (Rapa) for 1 h. Cells were then immunostained with a PolyE-tubulin antibody (red). Scale bar, 10 μm.
B. Quantification of normalized PolyE tubulin intensity at centrosomes of cells from (A). Data are presented as mean ± SD (green: DMSO; red: rapamycin). n = 59, 22, 45, 30, 39, and 45 cells from left to right; 3 independent experiments. Students' *t*-tests were performed, and *P* values are indicated.

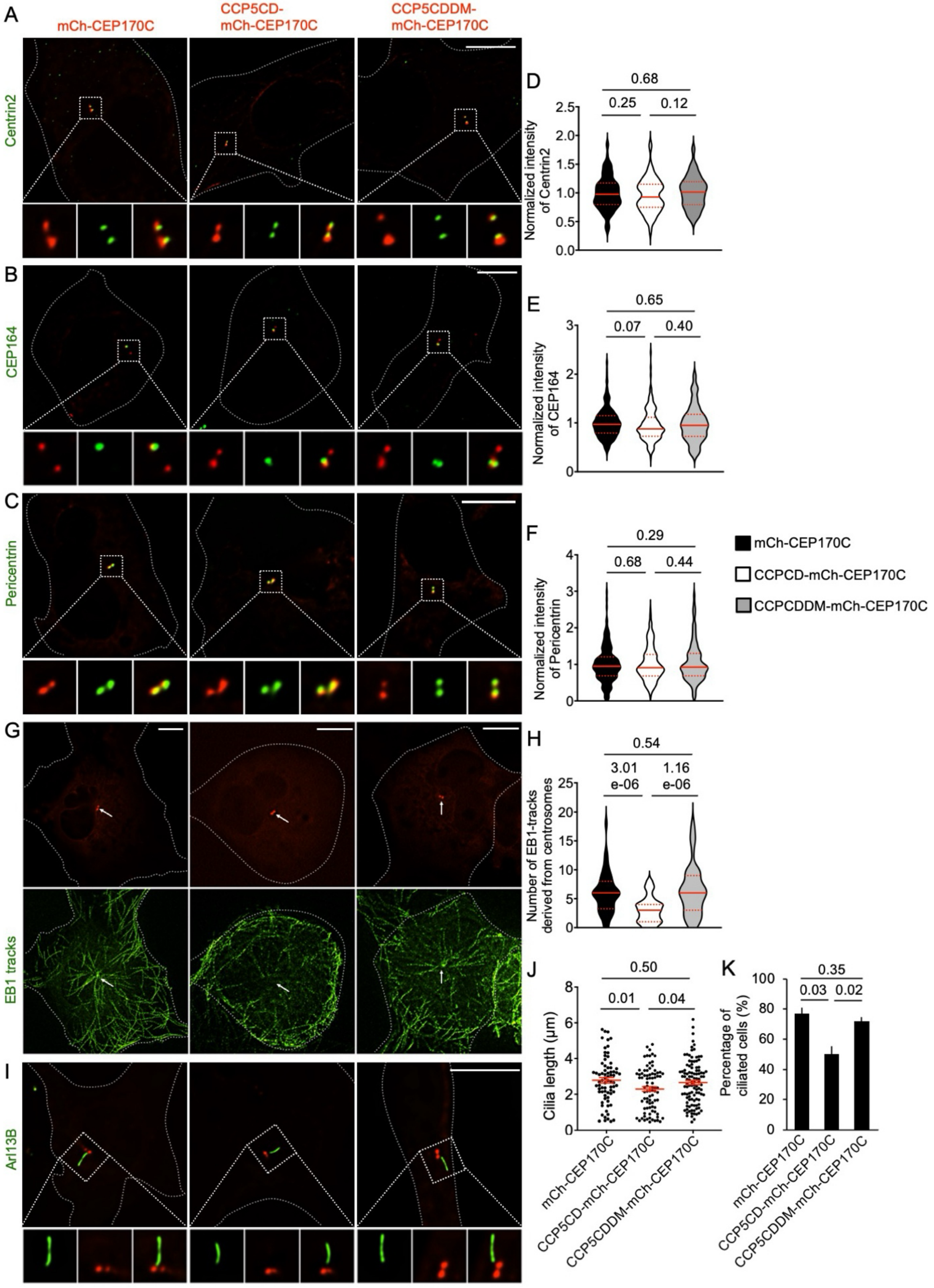
A
mCh-CEP170C
CCP5CD-
mCh-CEP170C
CCP5CDDM-
mCh-CEP170C
Centrin2
B
CEP164
C
Pericentrin
G
EB1 tracks
I
Arl13B
D
Normalized intensity of Centrin2
0.68
0.25
0.12
E
Normalized intensity of CEP164
0.65
0.07
0.40
F
Normalized intensity of Pericentrin
0.29
0.68
0.44
mCh-CEP170C
CCPCD-mCh-CEP170C
CCPCDDM-mCh-CEP170C
H
Number of EB1-tracks derived from centrosomes
0.54
3.01 e-06
1.16 e-06
J
Cilia length (µm)
0.50
0.01
0.04
K
Percentage of ciliated cells (%)
0.35
0.03
0.02
mCh-CEP170C
CCP5CD-mCh-CEP170C
CCP5CDDM-mCh-CEP170C

Appendix Figure S6. Long-term centrosomal hypoglutamylation suppresses microtuuble nucleation and cilia formation but has minimal impact on centrosome structure.

A-C. COS7 cells transfected with the indicated constructs were immunostained with the antibodies against Centrin2 (A), CEP164 (B), and Pericentrin (C), respectively. Lower panels show magnified images of the areas demarcated by the dashed squares. Scale bar, 10 μm.

D-F. The normalized intensity of Centrin2, CEP164, and Pericentrin of cells in (A-C), respectively. The solid red lines and dashed red lines in violin plots represent the median and first/third quartiles, respectively. n (from left to right) = 121, 122, and 116 cells in (D); 307, 210, and 115 cells in (E); 194, 248, and 194 cells in (F); 3~6 independent experiments.

G. COS7 cells were co-transfected with EB1-YFP and the indicated constructs. EB1-YFP comet trajectories were visualized using live-cell imaging. Maximun projections of EB1-YFP from 2.5-min imaging are shown. Arrows indicate centrosome regions. Scale bar, 10 μm.

H. The number of EB1-YFP tracks emitted from centrosomes in cells from (G) is shown. The solid red lines and dashed red lines in violin plots represent the median and first/third quartiles, respectively. n = 48, 68, 56 cells from left to right; 3 independent experiments.

I. NIH3T3 cells transfected with the indicated constructs were serum-starved for 24 h and then immunostained with Arl13 antibody (green) to visualize primary cilia. Scale bar, 10 μm.

J,K. Quantification of cilia length (J) and proportion of ciliated cells (K) in the cells shown in (I). Data are presented as mean ± S.E.M. n (from left to right) = 72, 82, and 114 cells in (J); from 3-6 independent experiments.

Students' *t*-tests were performed, and *P* values are indicated.

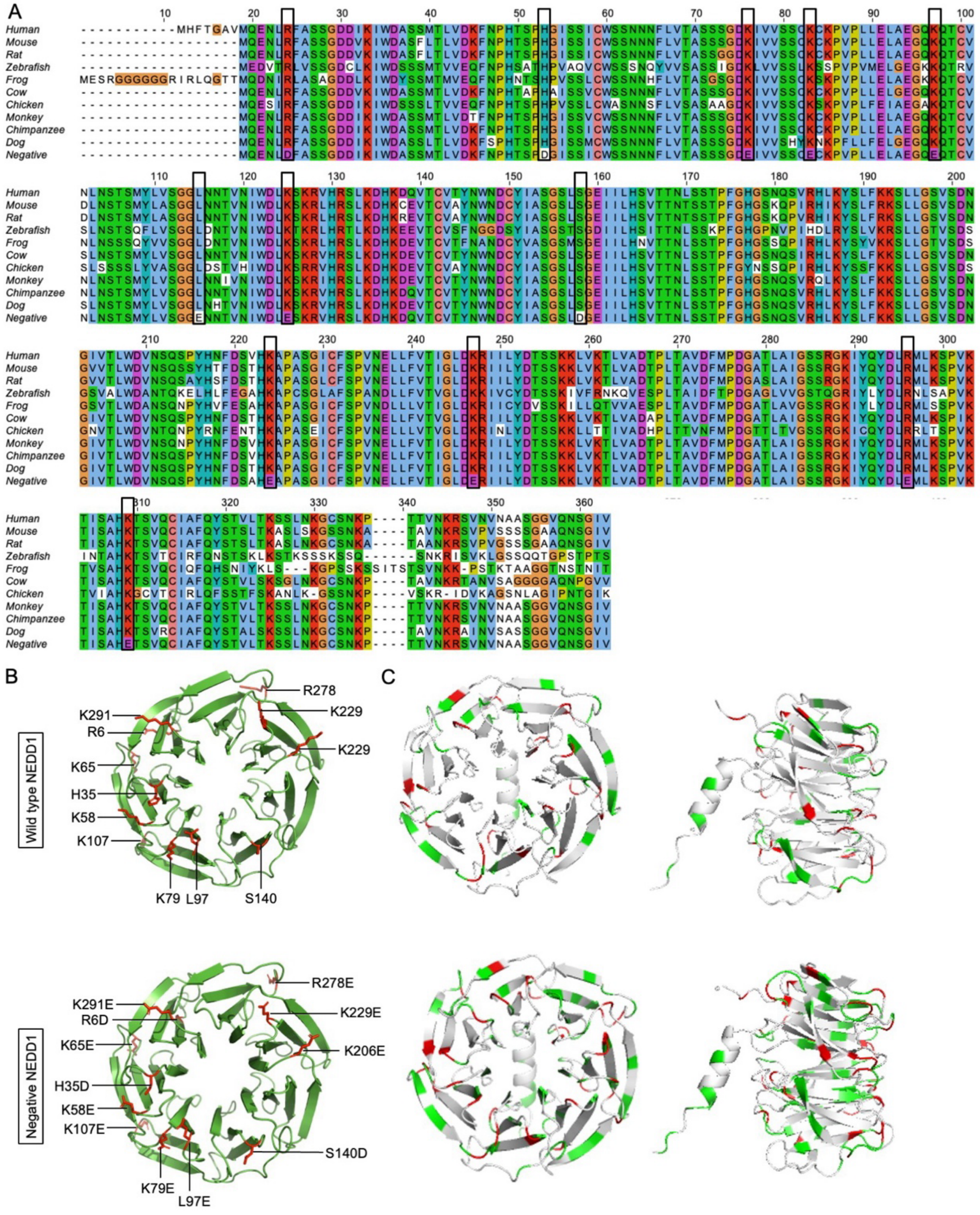

Appendix Figure S7. Modification of the NEDD1 centrosome-binding domain according to its charge

A. Alignment of NEDD1 centrosome-binding domains among 11 different species. The basic residues on the NEDD1 surface that are conserved among species were mutated to acidic residues (black outlines).

B. Centrosome-binding domain of wild-type NEDD1 and negative NEDD1. Several basic residues (green) on the NEDD1 surface were mutated to acidic residues (red).

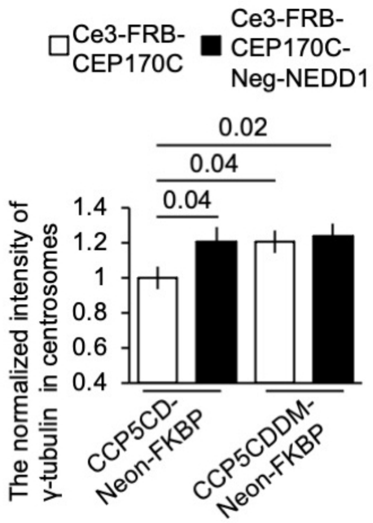

Appendix Figure S8. The charge of centrosome-binding domain in NEDD1 is not critical for γ-tubulin recruitment.

NIH3T3 cells were co-transfected with Ce3-FRB-CEP170C and CCP5CD-Neon-FKBP or with Ce3-FRB-CEP170C-neg-NEDD1 (a negatively charged NEDD1 mutant) and CCP5CDDM-Neon-FKBP. Transfected cells were incubated with 100 nM rapamycin (Rapa) for 1 h and then immunostained for γ-tubulin. The normalized intensity of γ-tubulin under the indicated conditions is presented as mean ± S.E.M. n = 37, 41, 61, and 52 cells from left to right; 3 independent experiments. Student's *t*-tests were performed, and *P* values are indicated.

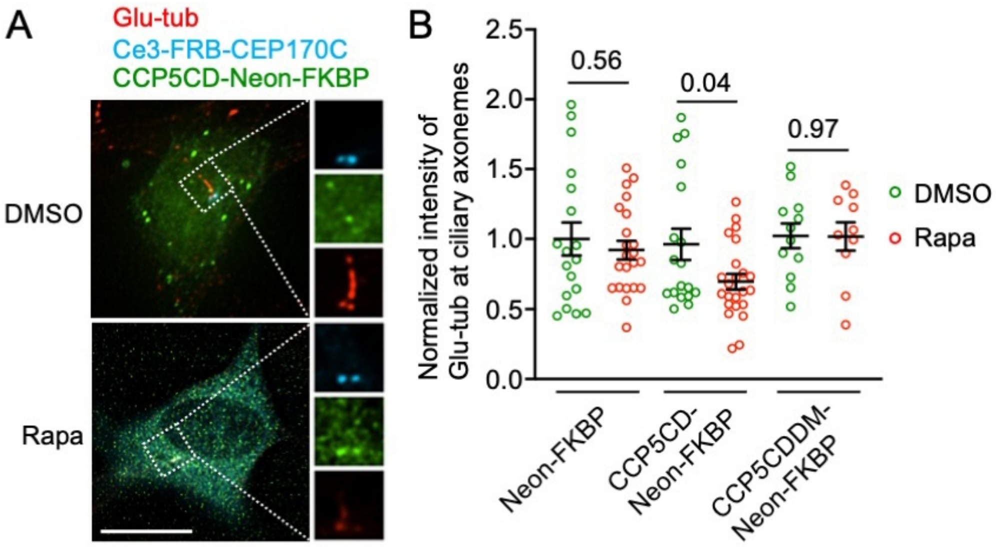

Appendix Figure S9. Hypoglutamylation at basal bod reduces axonemal glutamylation.

A. NIH3T3 cells co-transfected with Ce3-FRB-CEP170C (blue) and CCP5CD-Neon-FKBP (green) were pretreated with 100 nM rapamycin for 5 min. The treated cells were then serum-starved for 24 h and immunostained with the GT335 antibody (red). Scale bar, 10 μm.
B. Quantification of normalized glutamylated tubulin intensity at ciliary axonemes. Data represent the mean ± S.E.M. n = 18, 22, 18, 23, 12, and 10 cells from left to right; 3 independent experiment. Students' *t*-tests were performed, and *P* values are indicated.

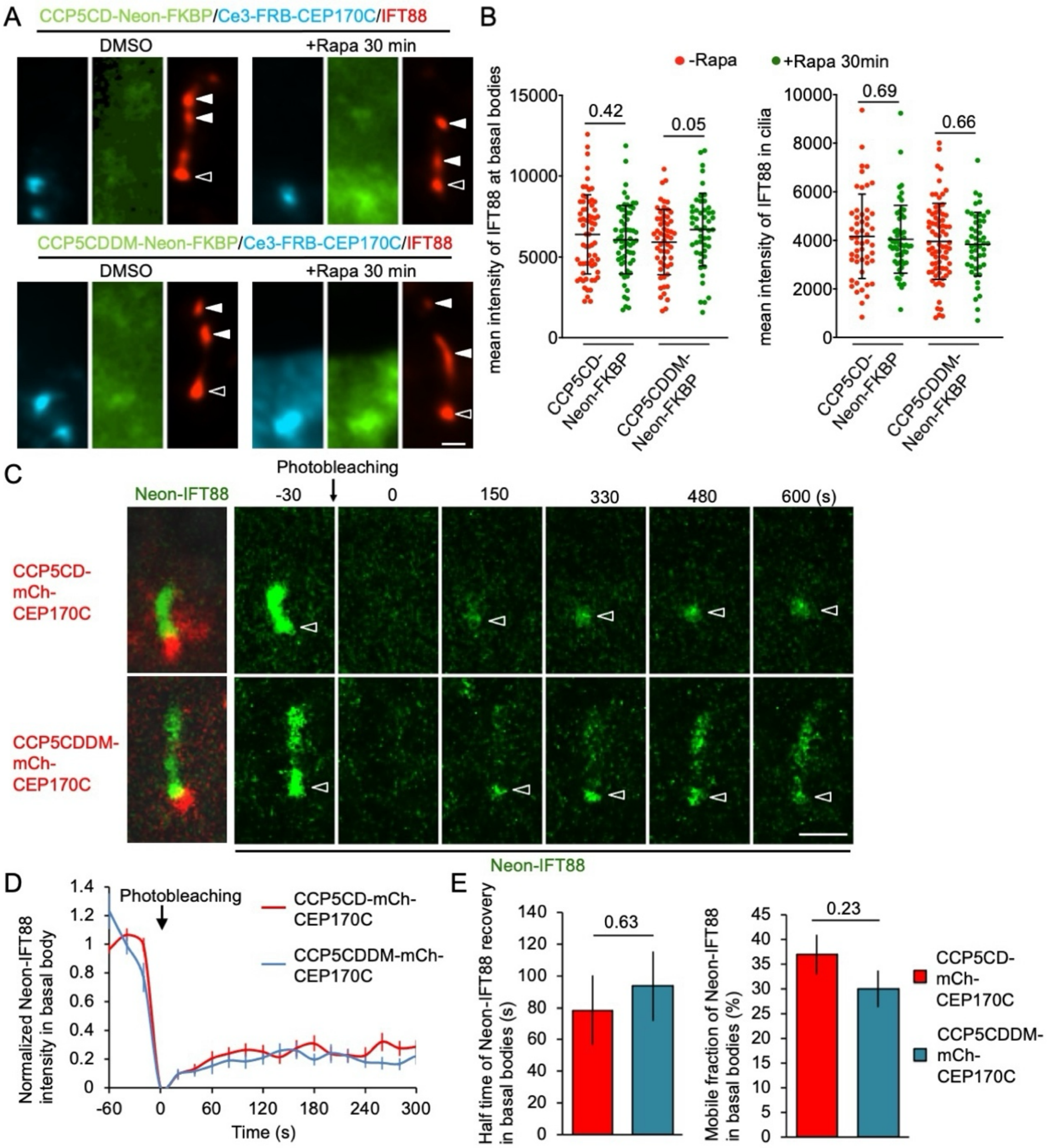

Appendix Figure S10. Acute glutamylation reduction at basal bodies does not affect docking and ciliary entry of the IFT machinery

A. NIH3T3 cells co-transfected with Ce3-FRB-CEP170C (blue) and either CCP5CD-Neon-FKBP or CCP5CDDM-Neon-FKBP (green) were serum-starved for 24 h and incubated with DMSO (0.1%) or rapamycin (100 nM) for 30 min. Cells were then immunostained for IFT88 (red). Open arrowheads and solid arrowheads indicate the IFT88 puncta in cilia and at the cilia base, respectively. Scale bar, 1 μm.

B. Mean fluorescence intensity of IFT88 at the ciliary base (open arrowheads in (A)) and in cilia (solid arrowheads in (A)). Data represent the mean ± S.E.M. n = 66, 62, 57, 50, 52, 55, 75, and 49 cells from left to right. Three independent experiments.

C. Neon-IFT88 fluorescence in NIH3T3 cells transfected with CCP5CD-mCh-CEP170C or CCP5CDDM-mCh-CEP170C was photobleached and then allowed to recover for the indicated times. Open arrowheads indicate the IFT88 signal at the cilia base. Scale bar, 1 μm.

D. Normalized fluorescence intensity of Neon-IFT88 in cells transfected with the indicated constructs in the experiment shown in (C). Data represent the mean ± S.E.M. n = 9 cells in each of the CCP5CD and CCP5CDDM groups. Four independent experiments.

E. Half-time of IFT88 recovery and mobile fraction of IFT88 in (C). Data represent the mean ± S.E.M. n = 9 cells in each of the CCP5CD and CCP5CDDM groups. Four independent experiments.

Student's *t*-tests were performed, and *P* values are indicated.

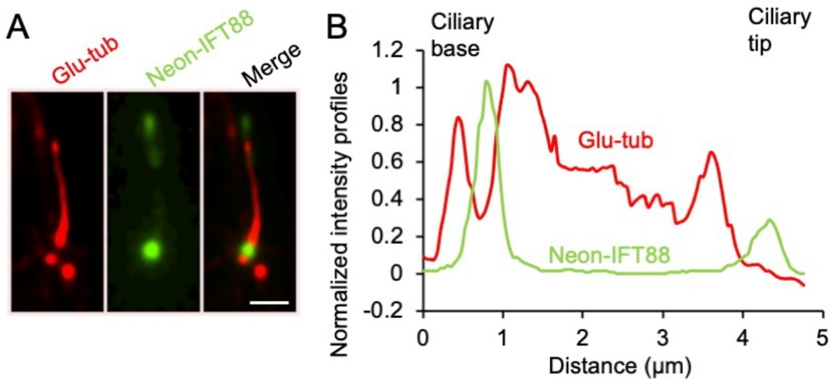

Appendix Figure S11. IFT88 does not colocalize with glutamylated sites of basal bodies

A. NIH3T3 cells transfected with Neon-IFT88 were serum-starved for 24 hƒ and then immunostained with antibody GT335. Scale bar, 1 µm.

B. Normalized Linescan profiles of Neon-IFT88 (green) and glutamylated tubulin (red) from the ciliary base to ciliary tip.

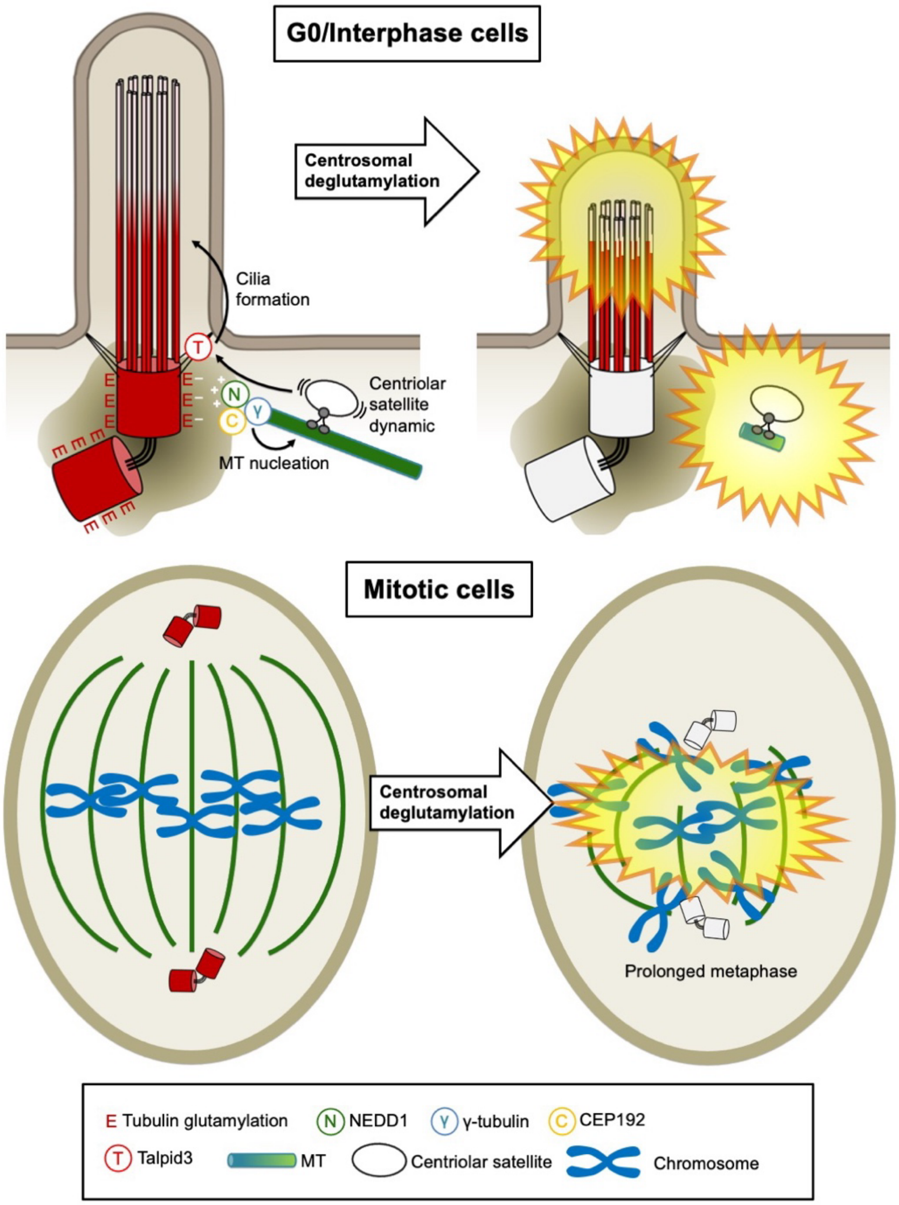

Appendix Figure S12. Working model depicting how glutamylation at centrosomes/basal bodies regulates cellular architecture and activities

Glutamylation physically recruits the NEDD1/CEP192/γ-tubulin complex via electrostatic forces for microtubule nucleation. Microtubule-derived from centrosomes serve as the railway to ensure centriolar satellite trafficking and promote ciliogenesis via Talpid3. During mitosis, centrosomal hypoglutamylation perturbs mitotic spindle formation and prolongs mitosis.

Key resources table

REAGENT or RESOURCE	SOURCE	IDENTIFIER
**Antibodies**		
Mouse monoclonal anti-α-tubulin	Sigma Aldrich	T6199
Mouse monoclonal anti-polyglutamylation modification, (GT335)	Adipogen	AG-20B-0020-C100
Mouse monoclonal anti-acetylated tubulin	Sigma Aldrich	T7451
Mouse monoclonal anti-Centrin2	Merck	04-1624
Rabbit polyclonal anti γ-tubulin	Sigma Aldrich	T6557
Rabbit polyclonal anti-NEDD1	Novus Biologicals	NBP1-83377
Rabbit polyclonal anti-IFT88	Proteintech	13967-1-AP
Rabbit polyclonal anti-CP110	Proteintech	12780-1-AP
Rabbit polyclonal anti-ARL13B	Proteintech	17711-1-AP
Rabbit polyclonal anti-tau	Dako	A0024
Rabbit polyclonal anti-PCM1	Proteintech	19856-1-AP
Rabbit polyclonal anti-CEP290	Abcam	ab84870
Rabbit polyclonal anti-Pericentrin	Abcam	ab220784
Rabbit polyclonal anti-Talpid3	Proteintech	24421-1-AP
Rabbit polyclonal anti-CEP164	Proteintech	22227-1-AP
Rabbit polyclonal anti-ODF2	Sigma Aldrich	HPA001874
Rat monoclonal anti-mCherry	ThermoFisher	M11217
Rabbit polyclonal anti CEP83	Gift from Dr. Wong-Jing Wang (National Yang Ming Chiao Tung University, Taipei, Taiwan)	N/A
Rabbit polyclonal anti CEP192	Proteintech	18832-1-AP
Rabbit polyclonal anti-Polyglutamate chain (polyE)	AdipoGen	AG-25B-0030
**Chemicals**		
Rapamycin	LC Laboratories	CAS#53123-88-9
RO-3306	Sigma-Aldrich	SML0569
Nocodazole	Sigma-Aldrich	M1404
Paclitaxel	Sigma-Aldrich	T7402
GA3-AM	Gift from Dr. Tasuku Ueno (University of Tokyo)	N/A
**Experimental Models: Cell Lines**		
HEK293T	ATCC	CRL-3216
NIH3T3	ATCC	CRL-1658
U2Os	ATCC	HTB-96
Hela	ATCC	CCL-2
**Recombinant DNA**		
Cerulean3-FRB-CEP170C	This work	
CCP5CD-Neon-FKBP	This work	
CCP5CDDM-Neon-FKBP	This work	
GFP-CEP170FL		
GFP-CEP170C	This work	

GFP-Centrin2	This work
GFP-Chibby	This work
GFP-CPAP	This work
GFP-Kizuna	This work
GAIs-CFP-CEP170C	This work
Neon-mGID1	This work
CCP5CD-mCherry-FKBP	This work
EB1-YFP	This work
Cerulean3-FRB-CEP170C-NEDD1	This work
Cerulean3-FRB-CEP170C-Neg-NEDD1	This work
PCM1F2-mCherry	This work
Cerulean3-FRB-CEP170C-Talpid3	This work
mCherry-FRB-CEP170C	This work
CCP5CD-mCherry-FKBP-P2A-FRB-CEP170C	This work
CCP5CDDM-mCherry-FKBP-P2A-FRB-CEP170C	This work
YFP-Centrin2	This work
mCherry-CEP170C	This work
CCP5CD-mCherry-CEP170C	This work
CCP5CDDM-mCherry-CEP170C	This work
Neon-IFT88	This work
CEP192-Cerulean3-FRB-CEP170C	This work
**Software and Algorithms**	
Nikon element AR software	Nikon
Prism	GraphPad
Zen	Zeiss
Huygens deconvolution	Scientific Volume Imaging
iBright™ FL1500 Instrument	Thermo Scientific
