# Supplementary material for: Glutamylation of centrosomes ensures their function by recruiting microtubule nucleation factors
Source: EMBO J. 2025 Apr 14;44(10):2976–96. doi: 10.1038/s44318-025-00435-y (PMC12084555; doi:10.1038/s44318-025-00435-y)
Supplement: Supplementary file 5 — Movie EV1 [file 44318_2025_435_MOESM5_ESM.zip › Movie EV1/Movie EV1.docx]

**Movie EV1.** **Rapid translocation of POIs to centrosomes**

NIH3T3 cells were co-transfected with Ce3-FRB-CEP170C and YFP-FKBP 1 day before imaging. Following the addition of 100 nM rapamycin, YFP-FKBP rapidly accumulated at centrosomes. Images were taken every 10 sec for 10 min. Scale bar, 10 µm. See also Fig 1B and 1C.
