# Supplementary material for: Glutamylation of centrosomes ensures their function by recruiting microtubule nucleation factors
Source: EMBO J. 2025 Apr 14;44(10):2976–96. doi: 10.1038/s44318-025-00435-y (PMC12084555; doi:10.1038/s44318-025-00435-y)
Supplement: Supplementary file 6 — Movie EV2 [file 44318_2025_435_MOESM6_ESM.zip › Movie EV2/Movie EV2.docx]

**Movie EV2. Using the gibberellin system to translocate POIs to centrosomes**

NIH3T3 cells were co-transfected with GAIs-CFP-CEP170C and Neon-mGID1 1 day before imaging. Following the addition of 100 µM GA3-AM, Neon-mGID1 rapidly accumulated at centrosomes. Images were captured every 10 sec for 10 min. Scale bar, 10 µm. See also Appendix Fig S4.
