# Supplementary material for: Glutamylation of centrosomes ensures their function by recruiting microtubule nucleation factors
Source: EMBO J. 2025 Apr 14;44(10):2976–96. doi: 10.1038/s44318-025-00435-y (PMC12084555; doi:10.1038/s44318-025-00435-y)
Supplement: Supplementary file 7 — Movie EV3 [file 44318_2025_435_MOESM7_ESM.zip › Movie EV3/Movie EV3.docx]

**Movie EV3. Dynamics of centriolar satellites upon centrosomal hypoglutamylation**

COS7 cells co-transfected with Ce3-FRB (CR)-CEP170C, CCP5CD-Neon-FKBP (NF), and PCM1F2-mCh (a centriolar satellite marker) and treated with rapamycin (100 nM) to induce centrosomal hypoglutamylation. Images were captured every 1 min for 125 min. Scale bar, 10 µm. See also Fig 5E.
