# Supplementary figures and images for: Glutamylation of centrosomes ensures their function by recruiting microtubule nucleation factors
Source: EMBO J. 2025 Apr 14;44(10):2976–96. doi: 10.1038/s44318-025-00435-y (PMC12084555; doi:10.1038/s44318-025-00435-y)

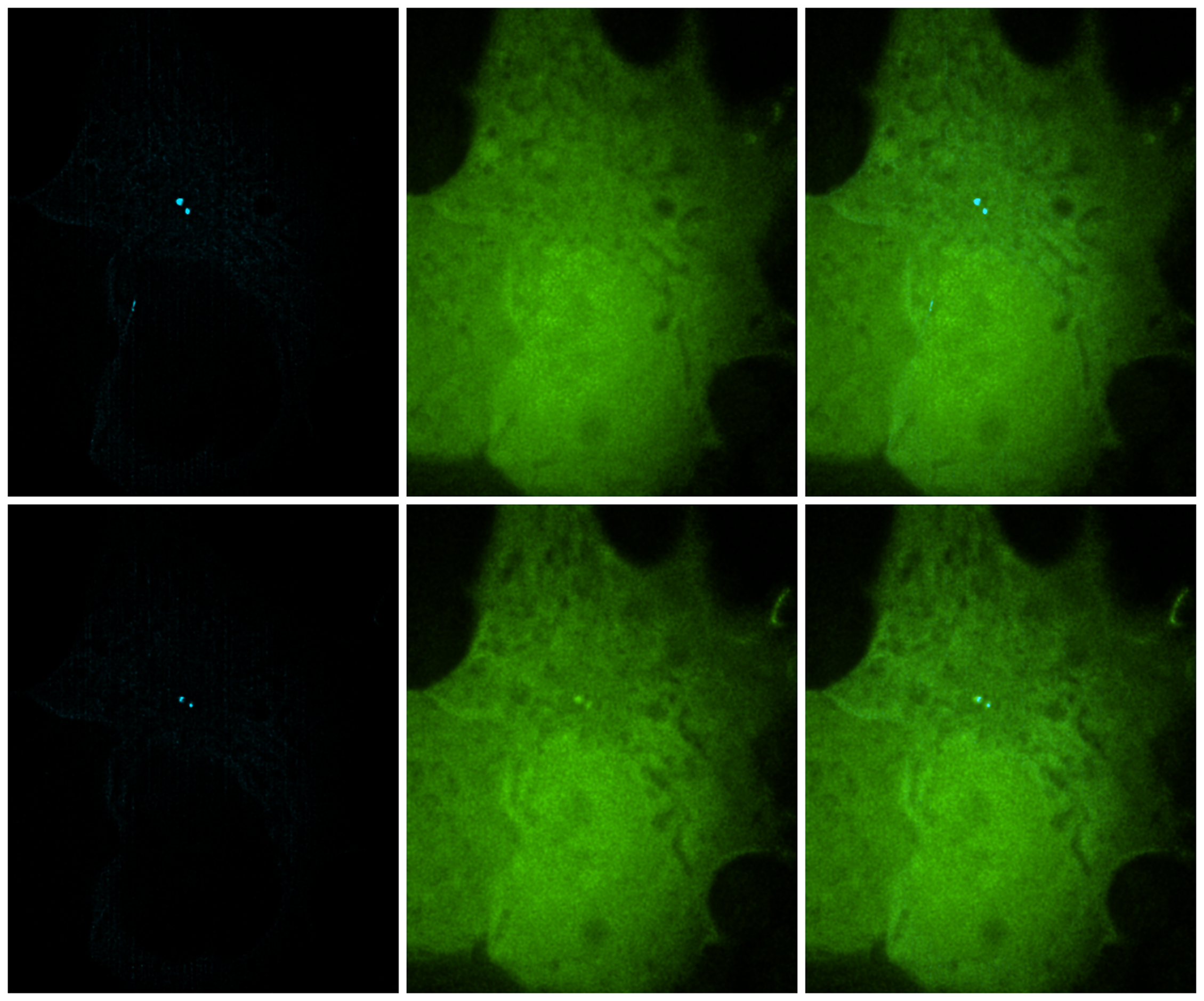

Supplement: Supplementary file 8 — Source data Fig. 1 [file 44318_2025_435_MOESM8_ESM.zip › SD Figure 1/1B.png]

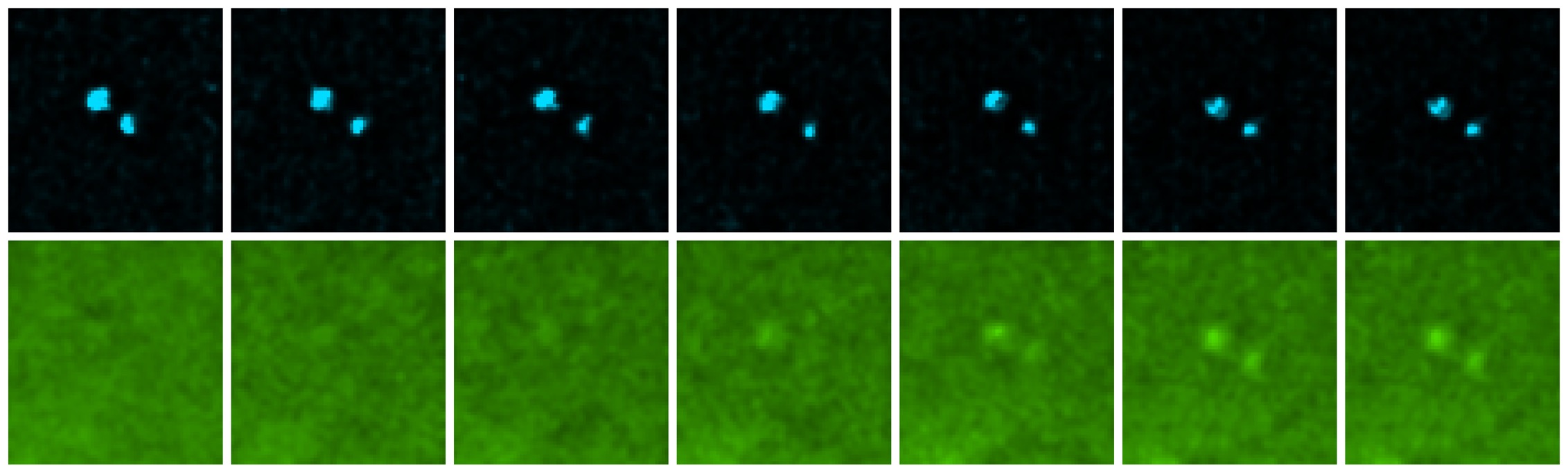

Supplement: Supplementary file 8 — Source data Fig. 1 [file 44318_2025_435_MOESM8_ESM.zip › SD Figure 1/1C.jpg]

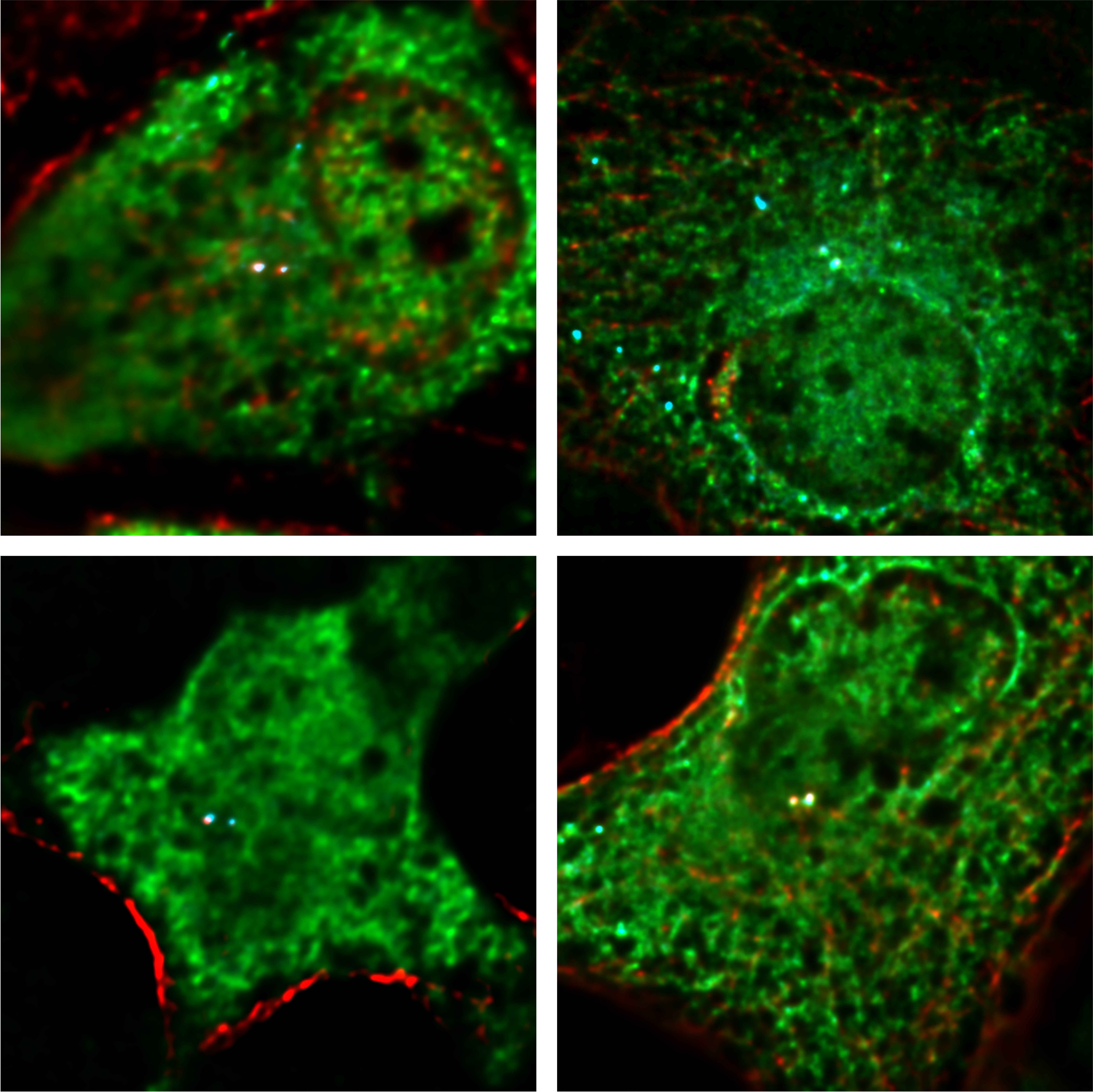

Supplement: Supplementary file 8 — Source data Fig. 1 [file 44318_2025_435_MOESM8_ESM.zip › SD Figure 1/1E.jpg]

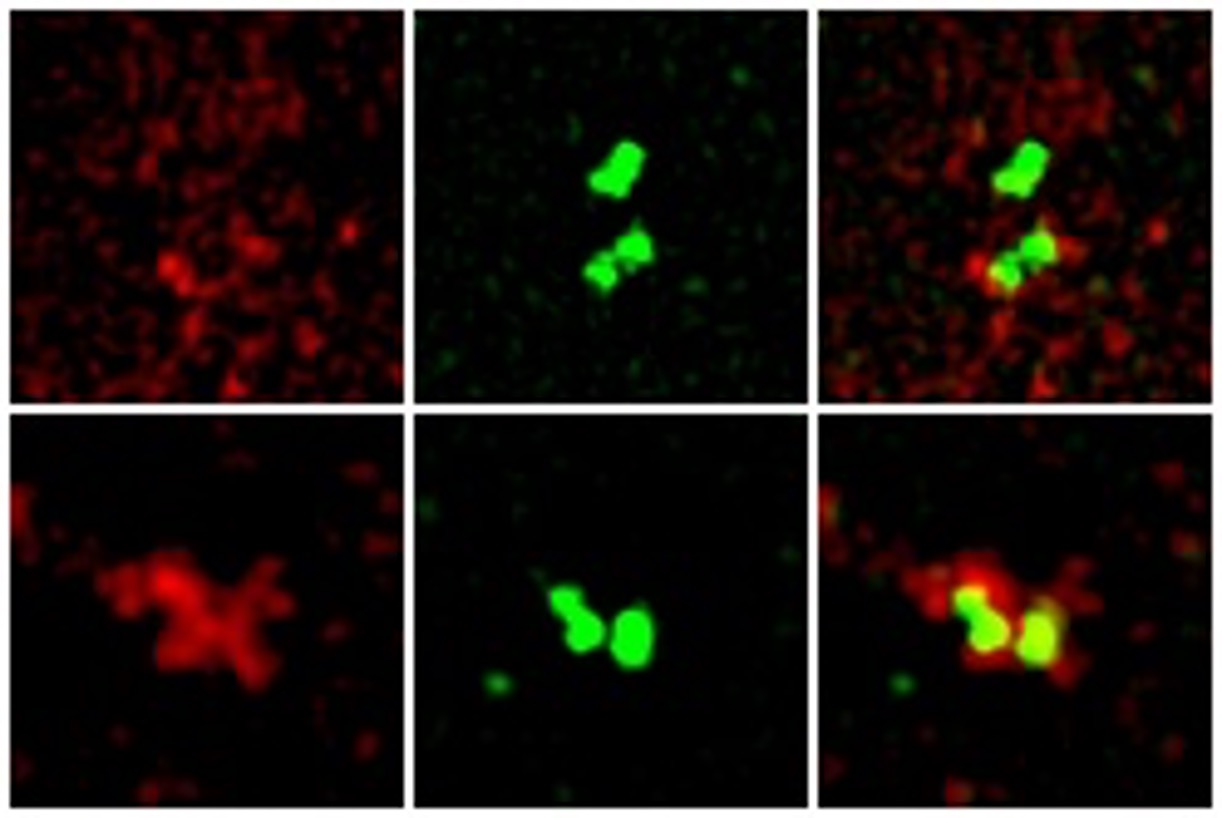

Supplement: Supplementary file 9 — Source data Fig. 2 [file 44318_2025_435_MOESM9_ESM.zip › SD Figure 2/2K.jpg]

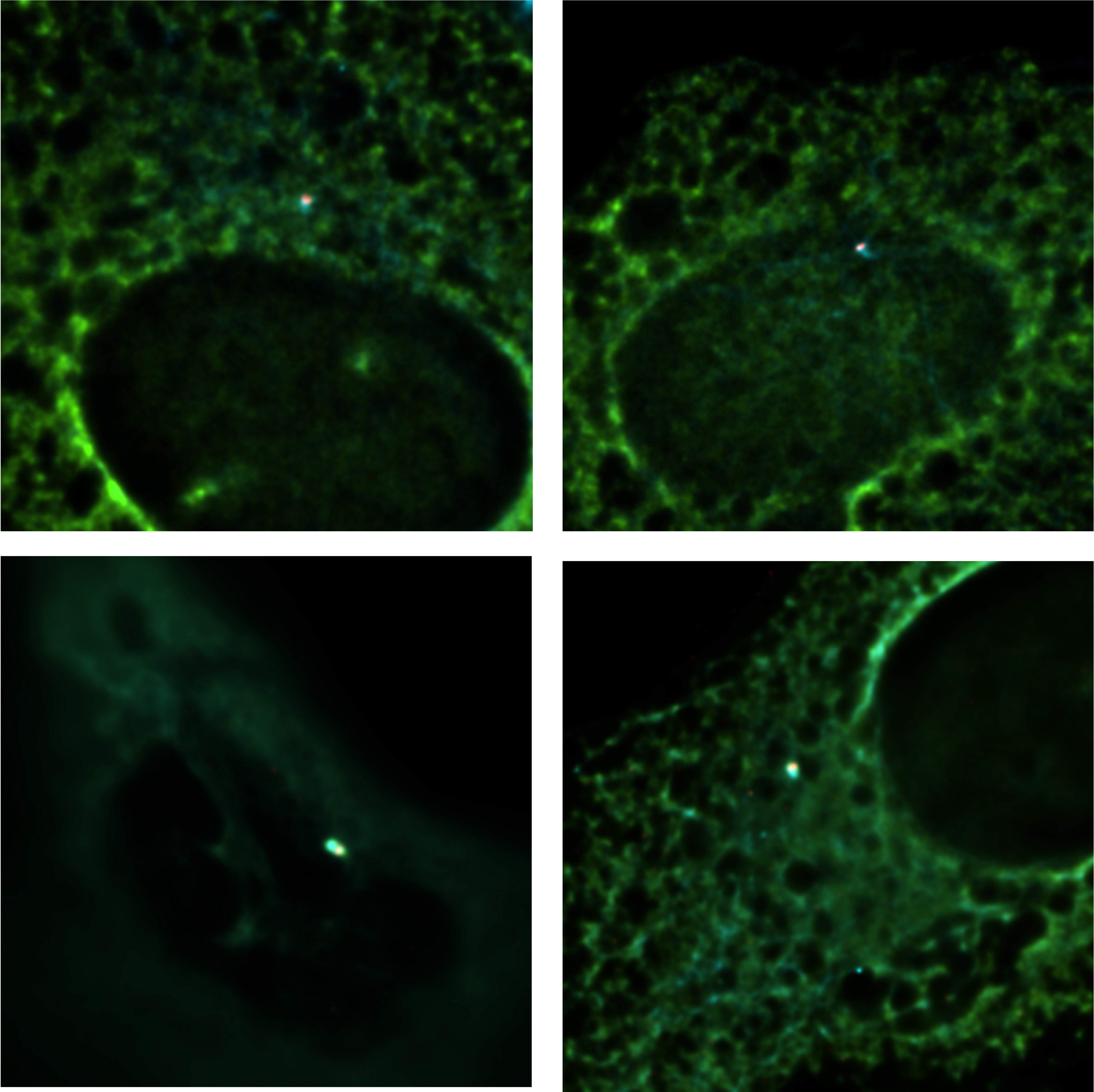

Supplement: Supplementary file 9 — Source data Fig. 2 [file 44318_2025_435_MOESM9_ESM.zip › SD Figure 2/2D.jpg]

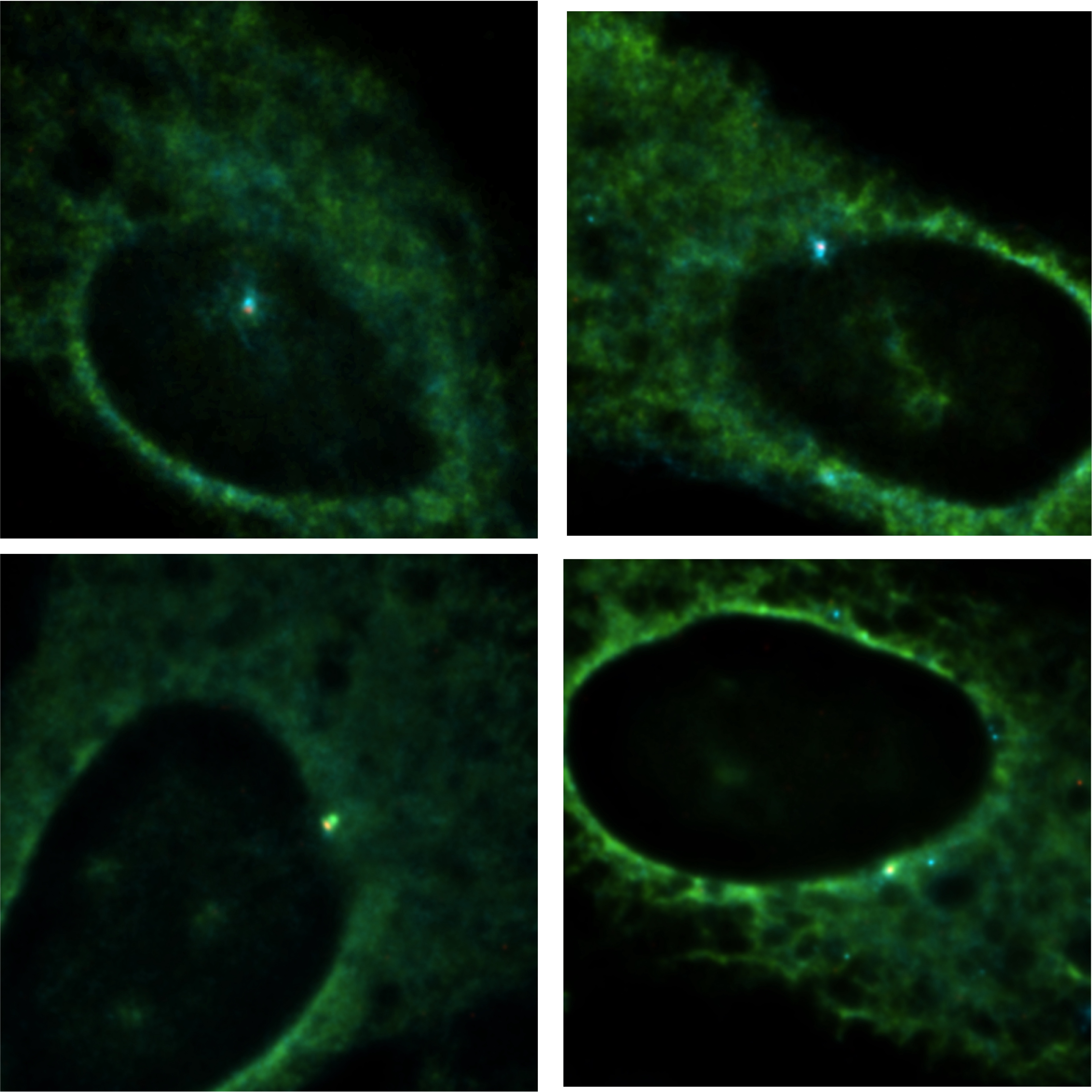

Supplement: Supplementary file 9 — Source data Fig. 2 [file 44318_2025_435_MOESM9_ESM.zip › SD Figure 2/2E.jpg]

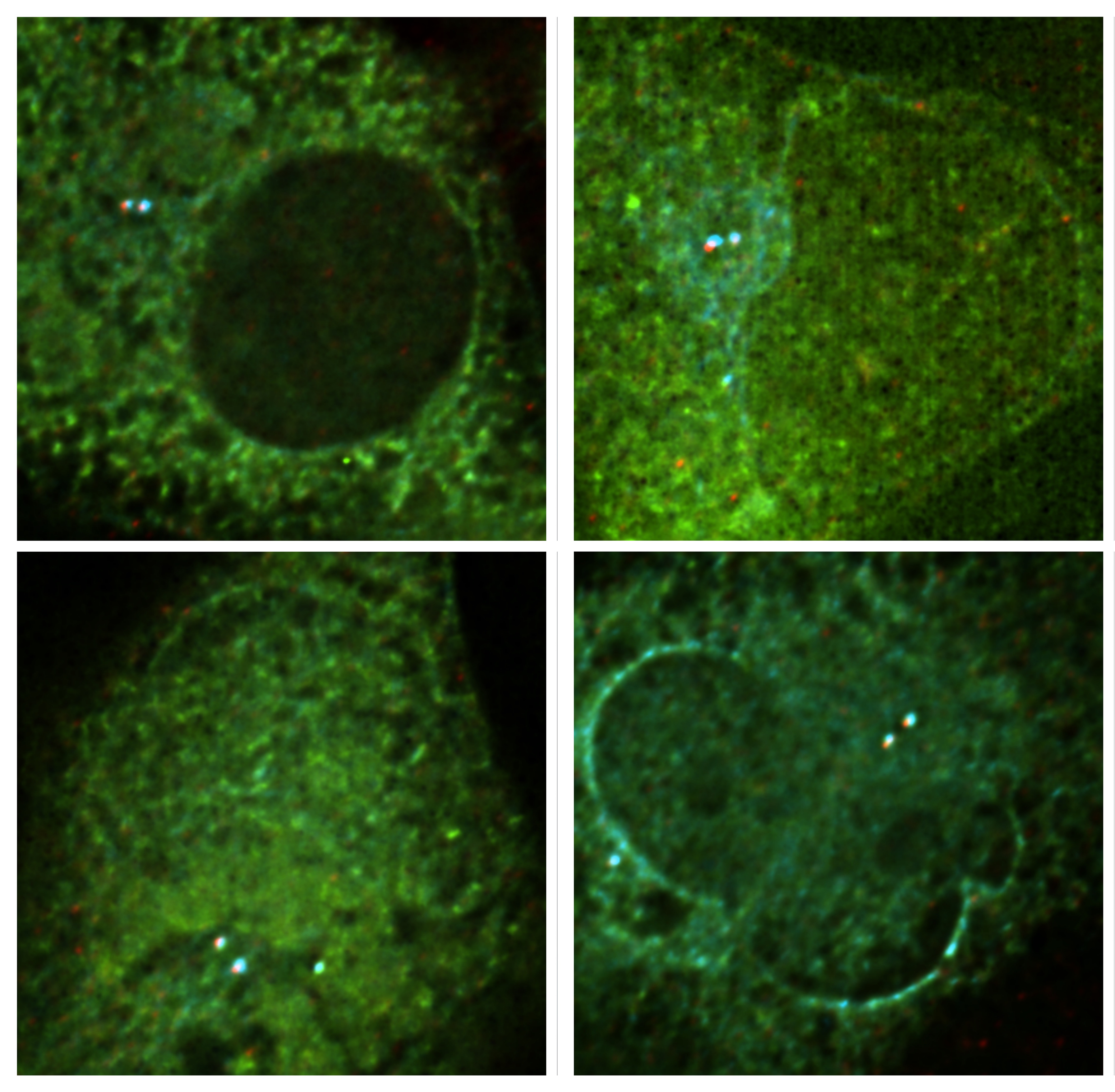

Supplement: Supplementary file 9 — Source data Fig. 2 [file 44318_2025_435_MOESM9_ESM.zip › SD Figure 2/2A.jpg]

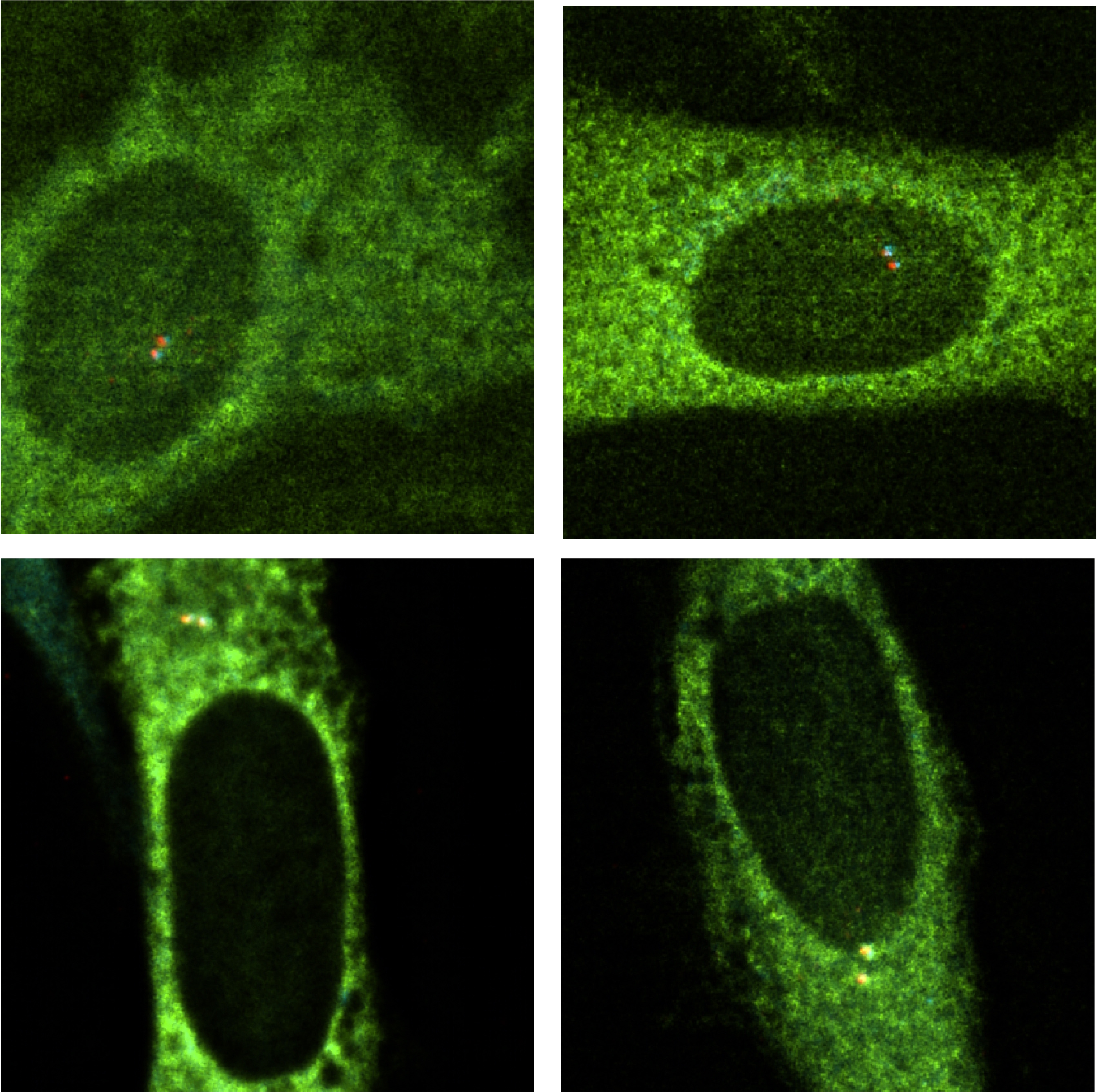

Supplement: Supplementary file 9 — Source data Fig. 2 [file 44318_2025_435_MOESM9_ESM.zip › SD Figure 2/2B.jpg]

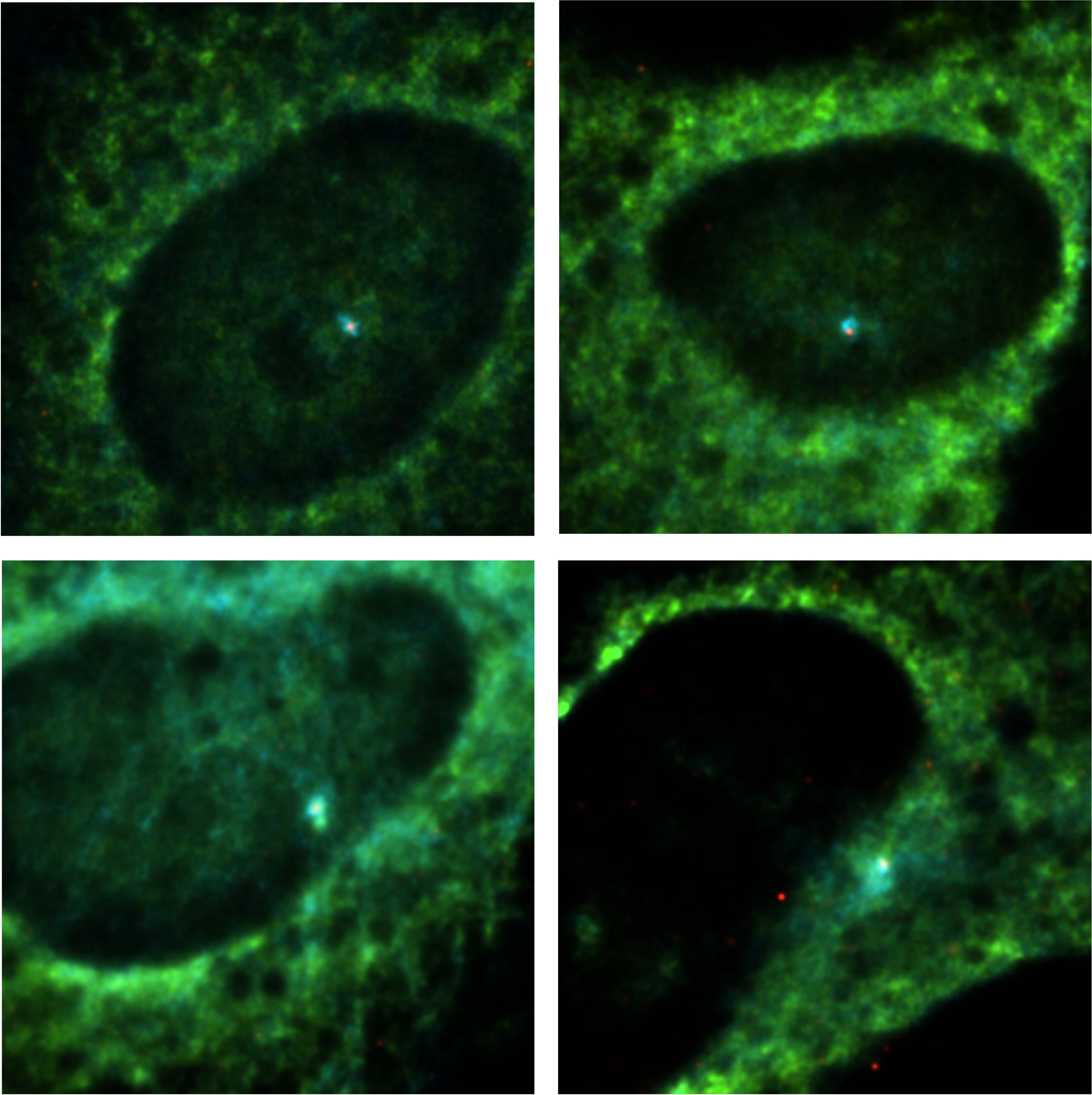

Supplement: Supplementary file 9 — Source data Fig. 2 [file 44318_2025_435_MOESM9_ESM.zip › SD Figure 2/2C.jpg]

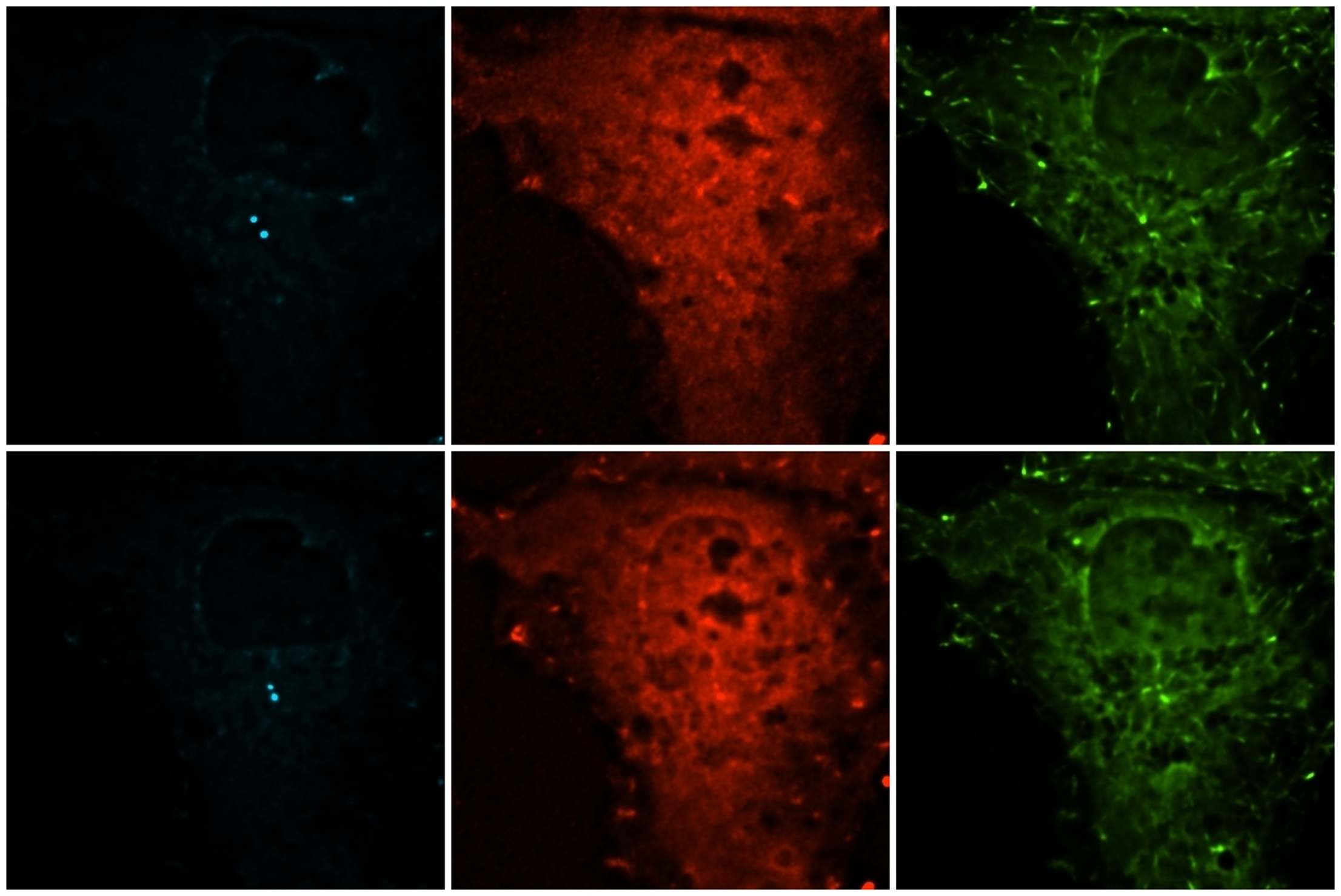

Supplement: Supplementary file 10 — Source data Fig. 3 [file 44318_2025_435_MOESM10_ESM.zip › SD Figure 3/3C.png]

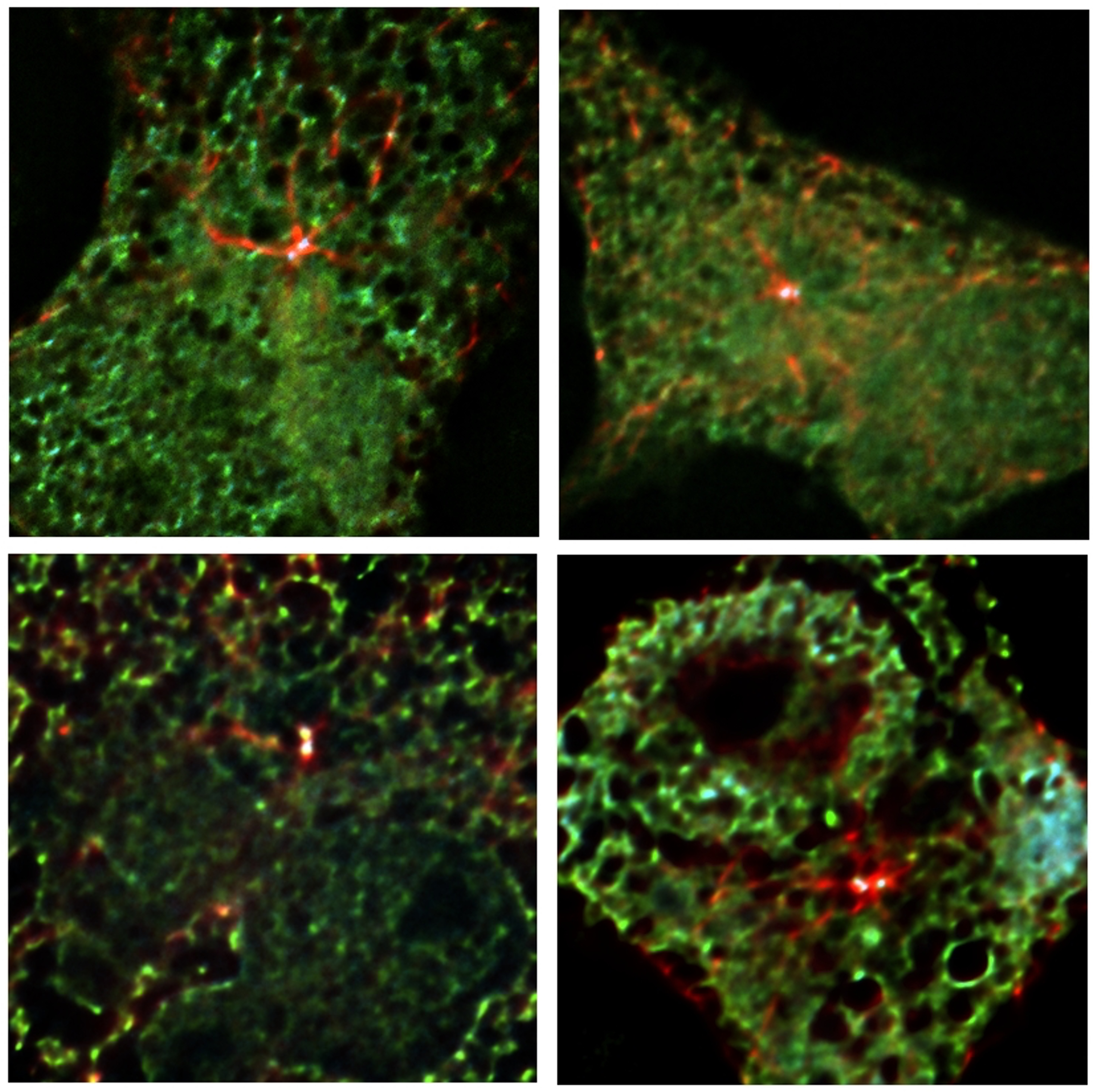

Supplement: Supplementary file 10 — Source data Fig. 3 [file 44318_2025_435_MOESM10_ESM.zip › SD Figure 3/3A.jpg]

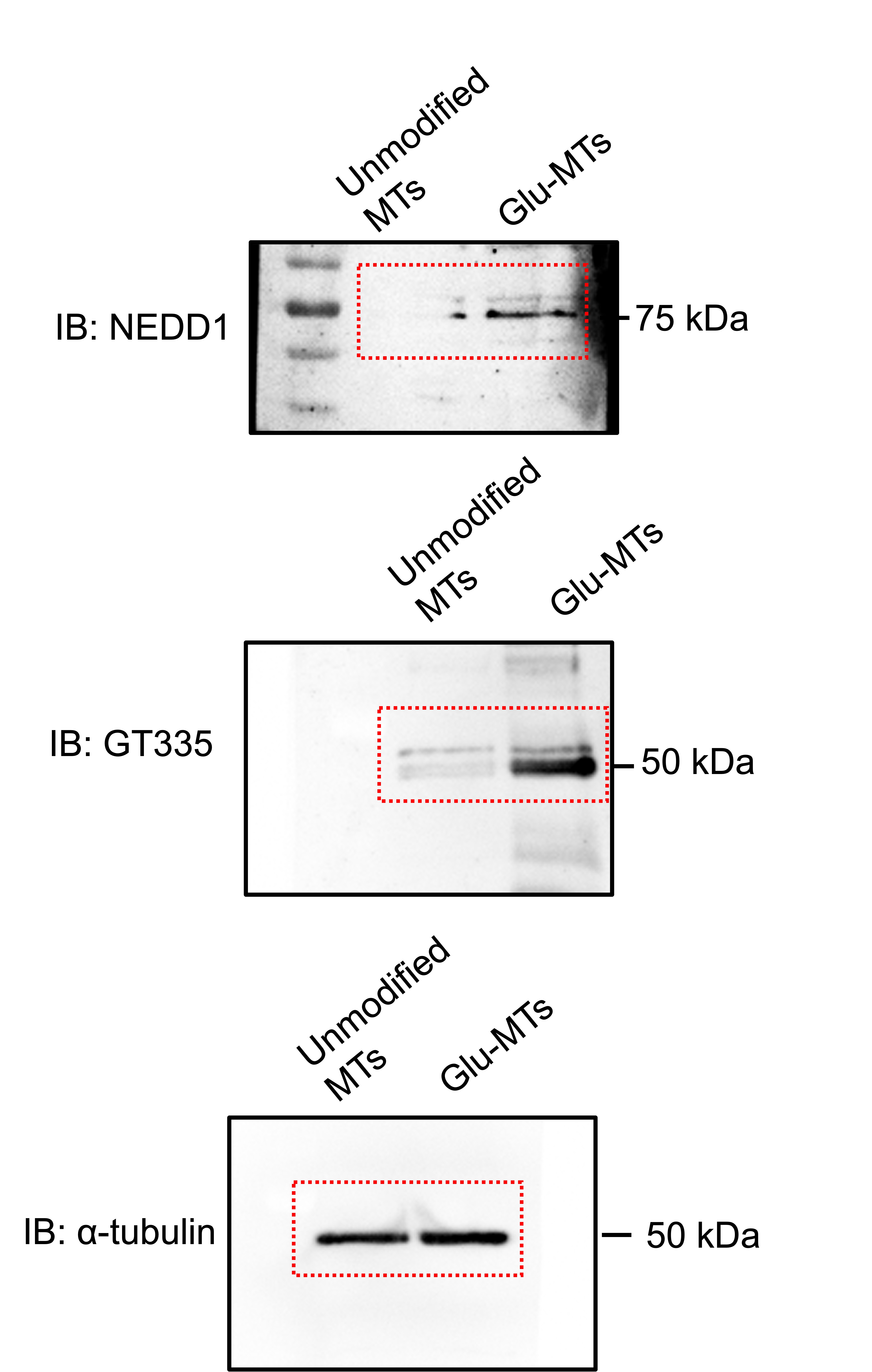

Supplement: Supplementary file 11 — Source data Fig. 4 [file 44318_2025_435_MOESM11_ESM.zip › SD Figure 4/4L.jpg]

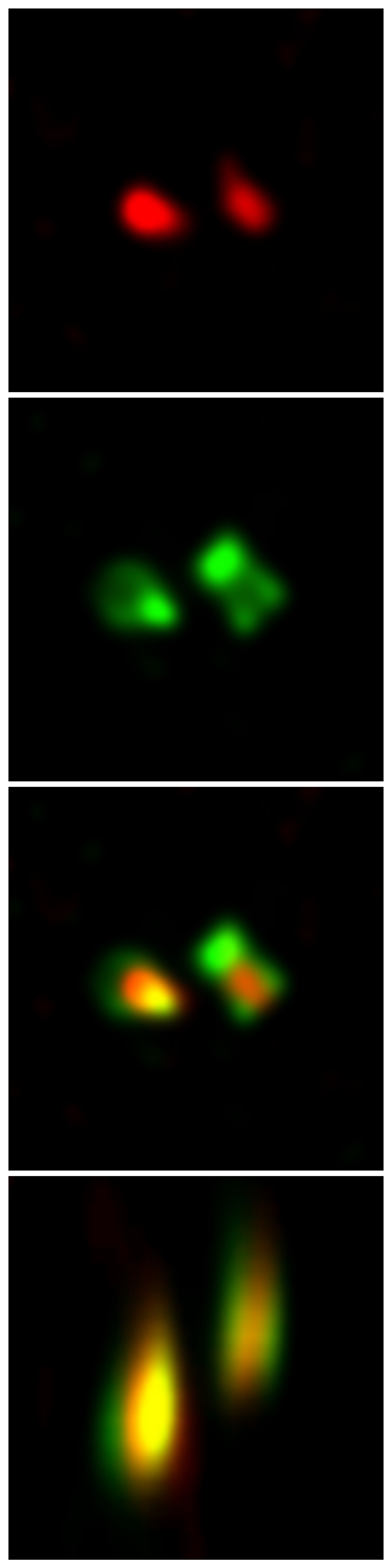

Supplement: Supplementary file 11 — Source data Fig. 4 [file 44318_2025_435_MOESM11_ESM.zip › SD Figure 4/4N.jpg]

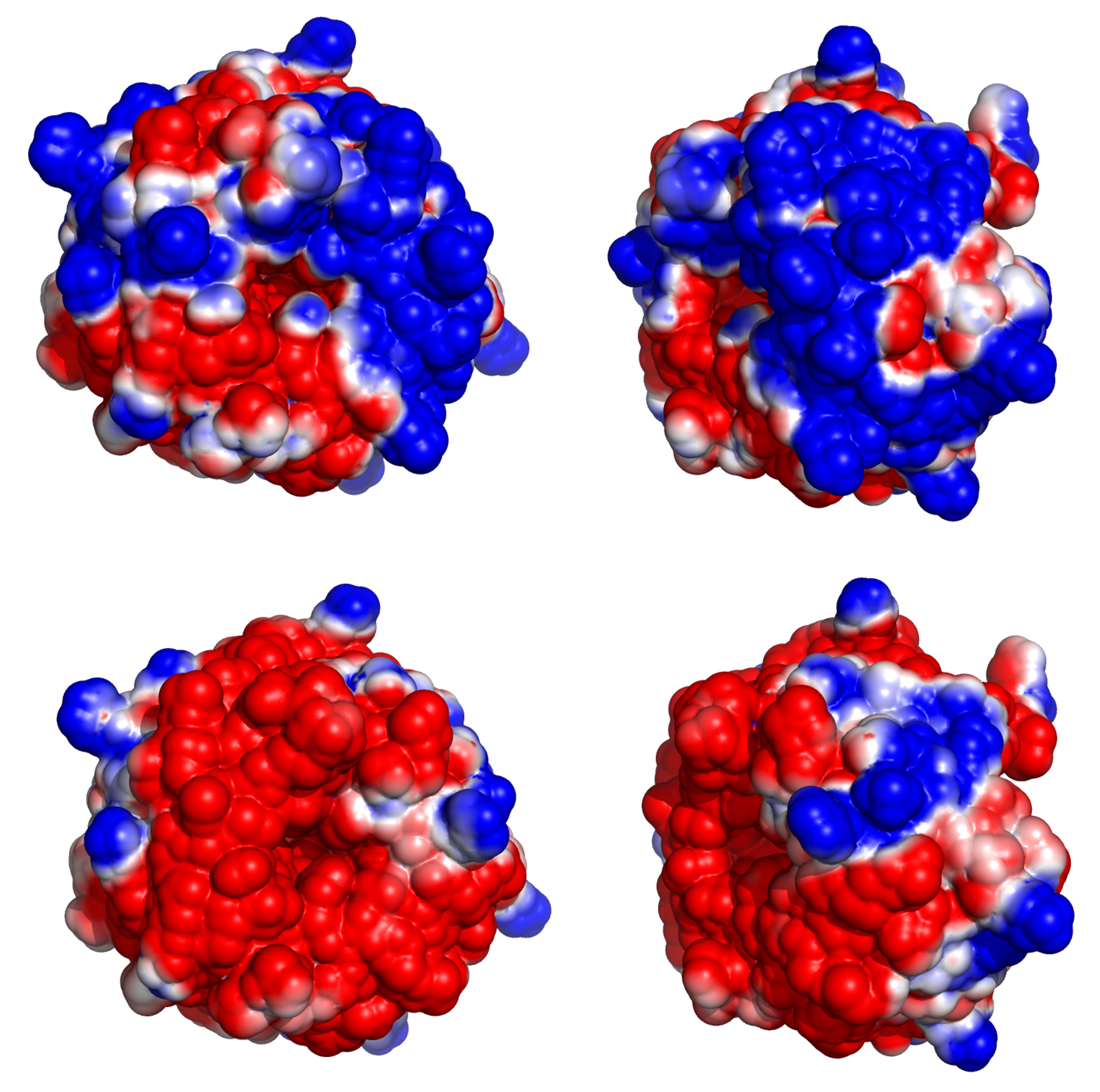

Supplement: Supplementary file 11 — Source data Fig. 4 [file 44318_2025_435_MOESM11_ESM.zip › SD Figure 4/4O.jpg]

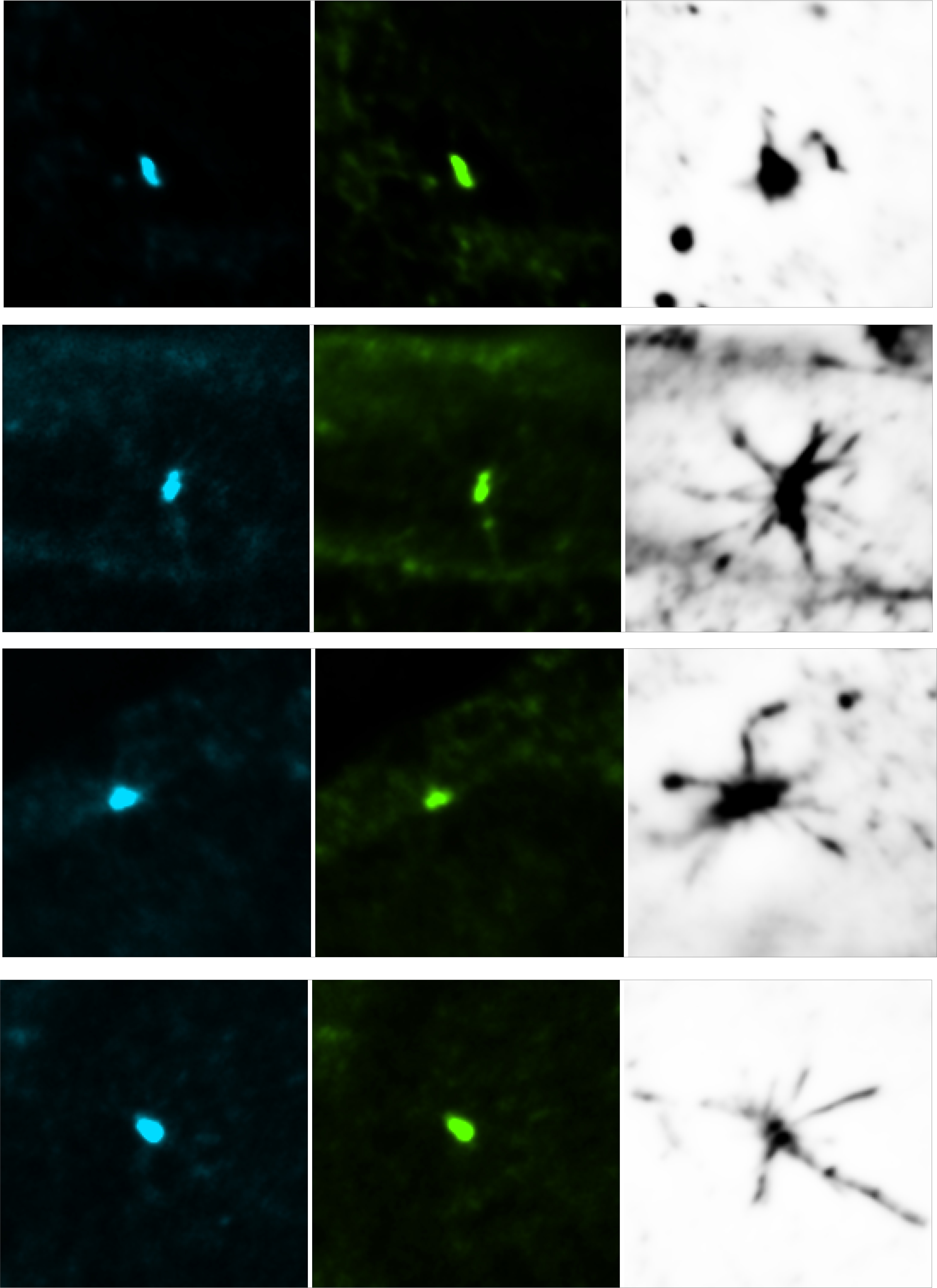

Supplement: Supplementary file 11 — Source data Fig. 4 [file 44318_2025_435_MOESM11_ESM.zip › SD Figure 4/4H.jpg]

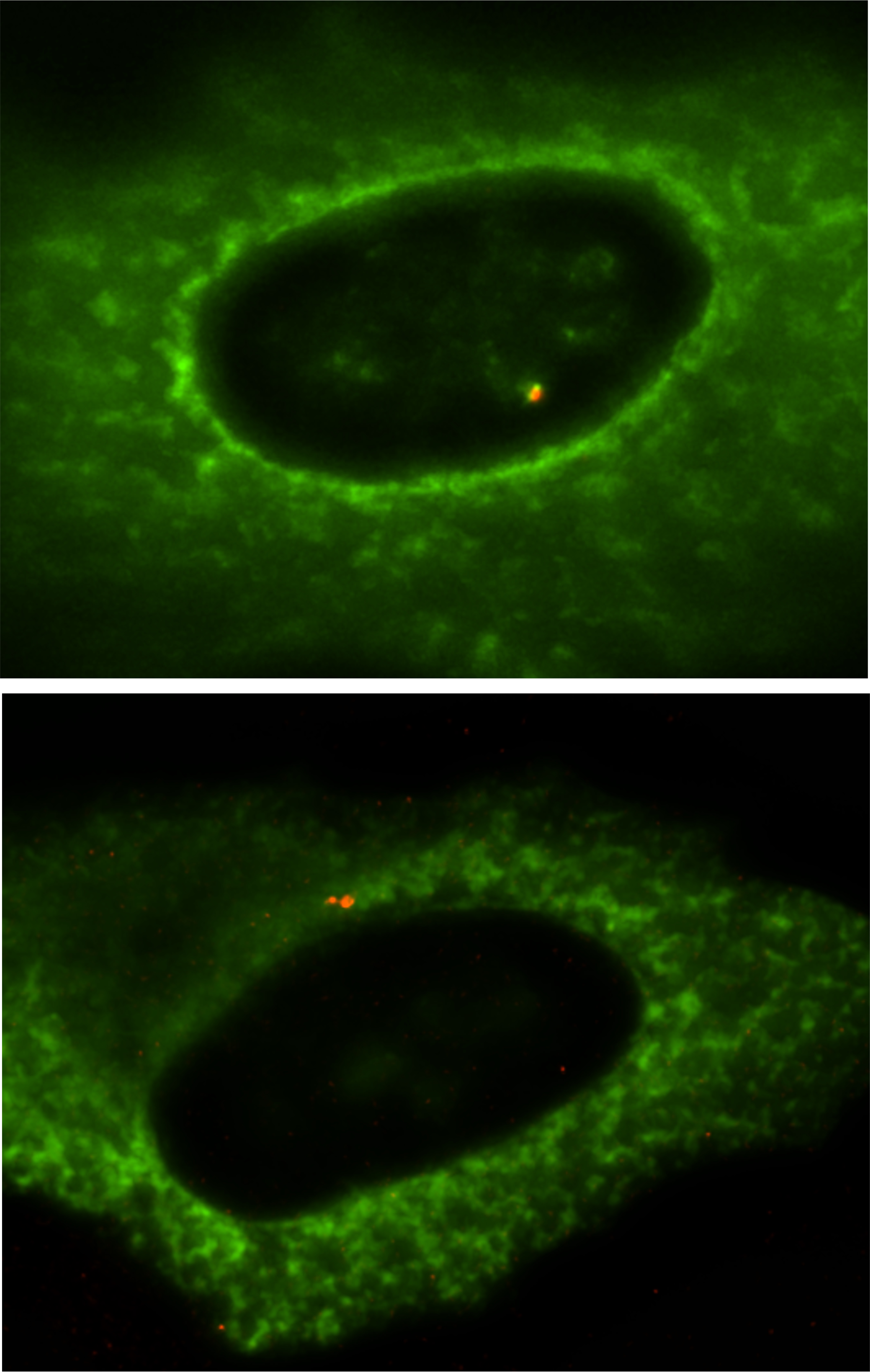

Supplement: Supplementary file 11 — Source data Fig. 4 [file 44318_2025_435_MOESM11_ESM.zip › SD Figure 4/4P.jpg]

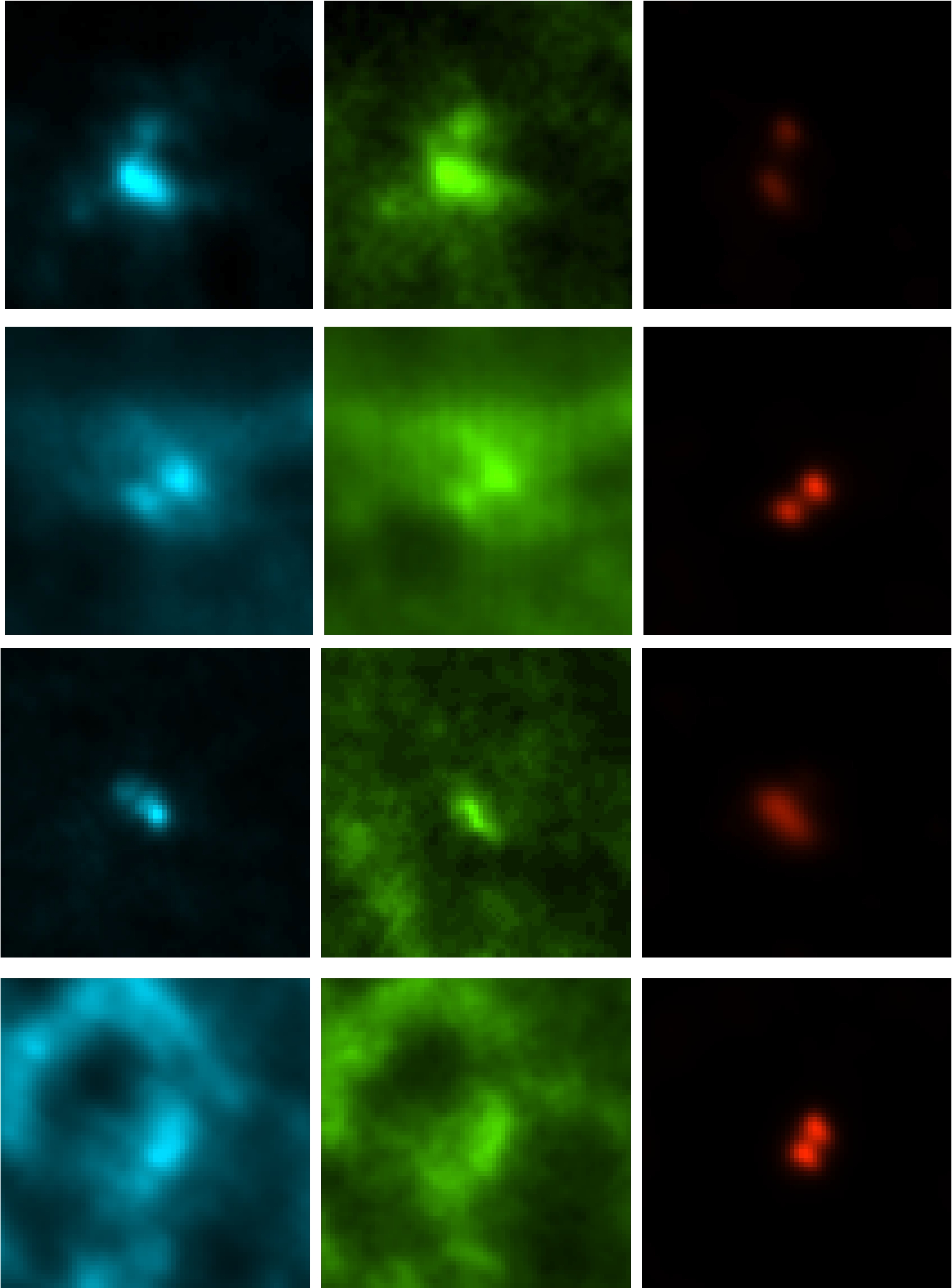

Supplement: Supplementary file 11 — Source data Fig. 4 [file 44318_2025_435_MOESM11_ESM.zip › SD Figure 4/4G.jpg]

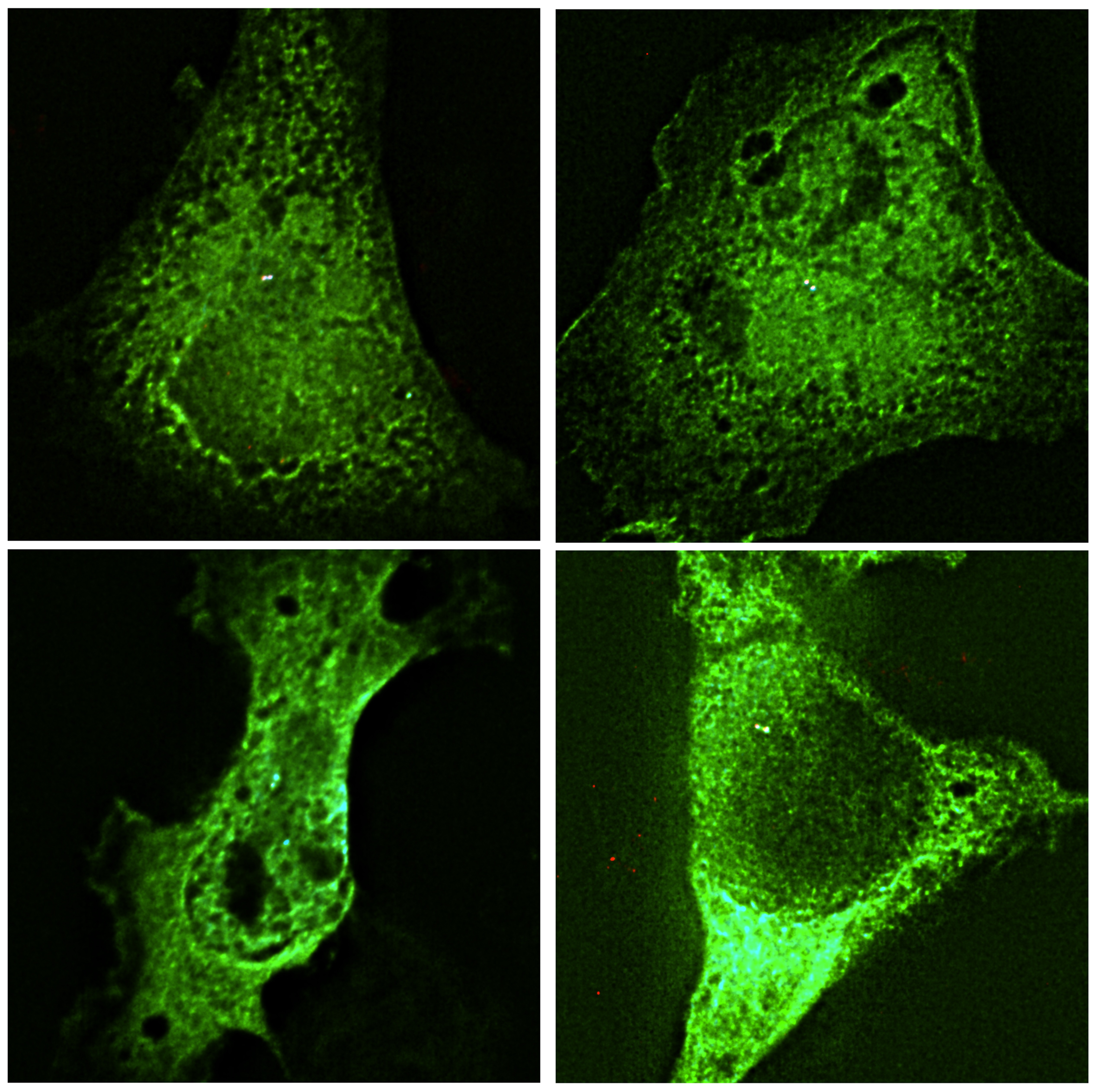

Supplement: Supplementary file 11 — Source data Fig. 4 [file 44318_2025_435_MOESM11_ESM.zip › SD Figure 4/4B.jpg]

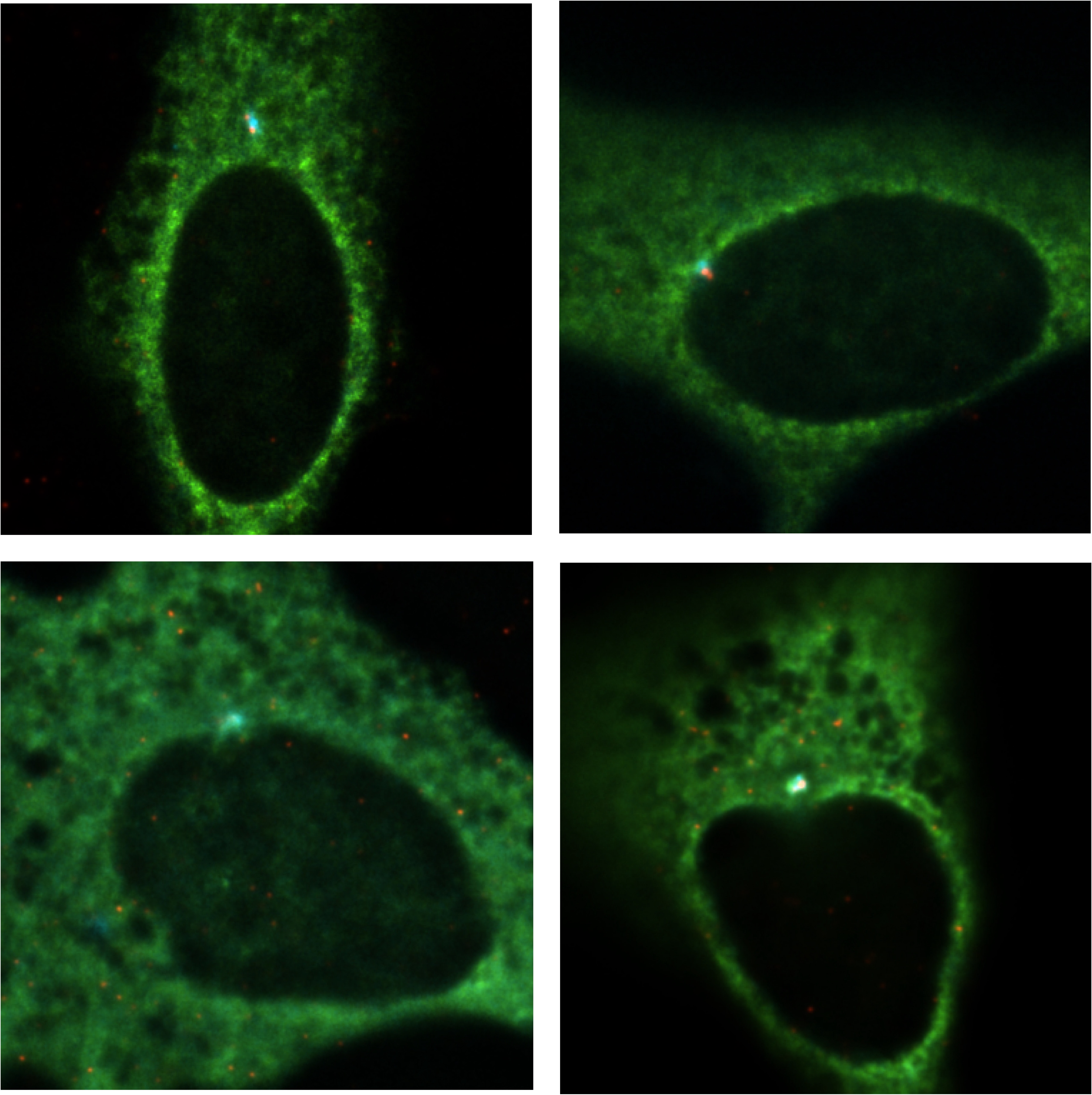

Supplement: Supplementary file 11 — Source data Fig. 4 [file 44318_2025_435_MOESM11_ESM.zip › SD Figure 4/4C.jpg]

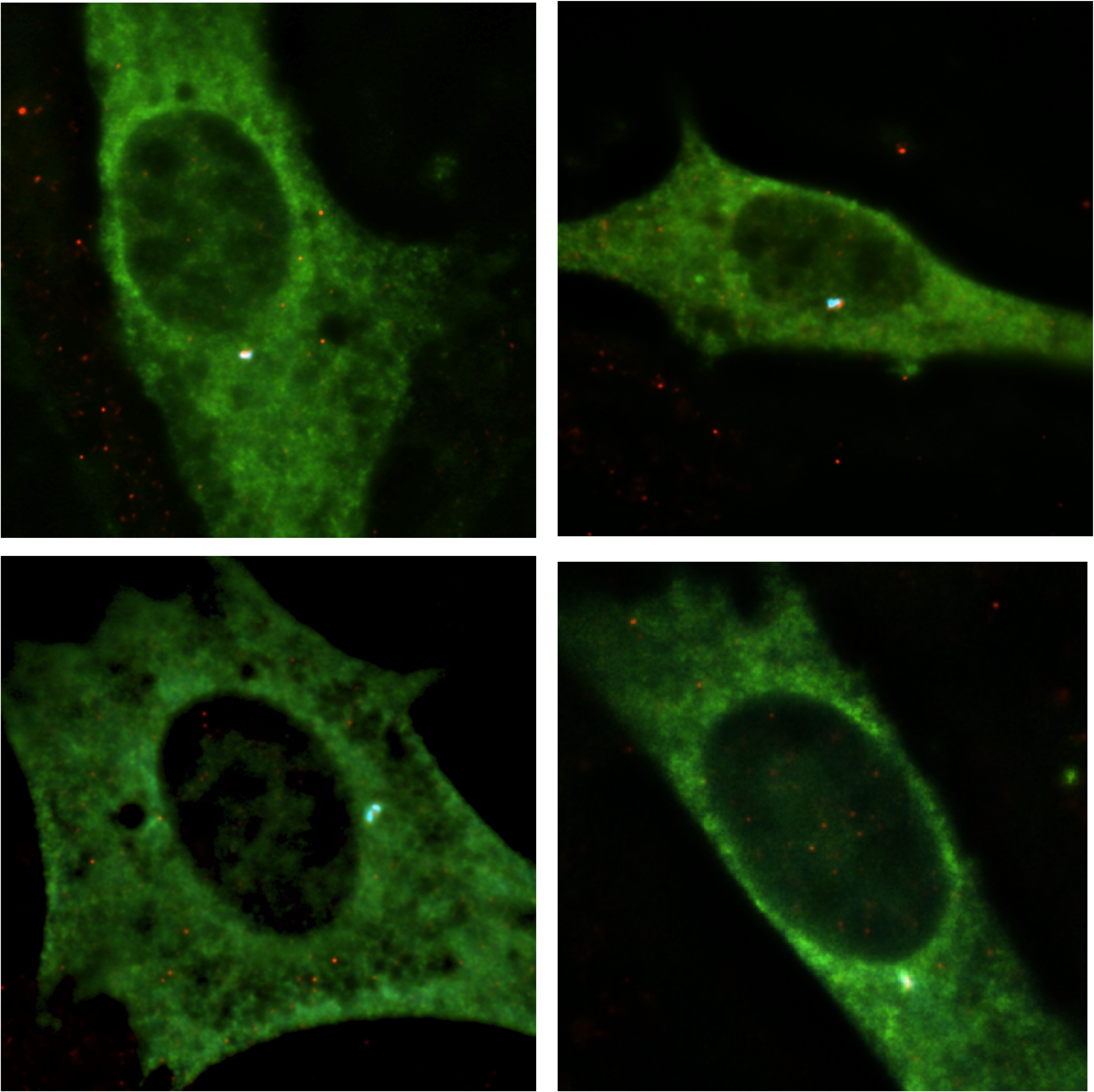

Supplement: Supplementary file 11 — Source data Fig. 4 [file 44318_2025_435_MOESM11_ESM.zip › SD Figure 4/4A.jpg]

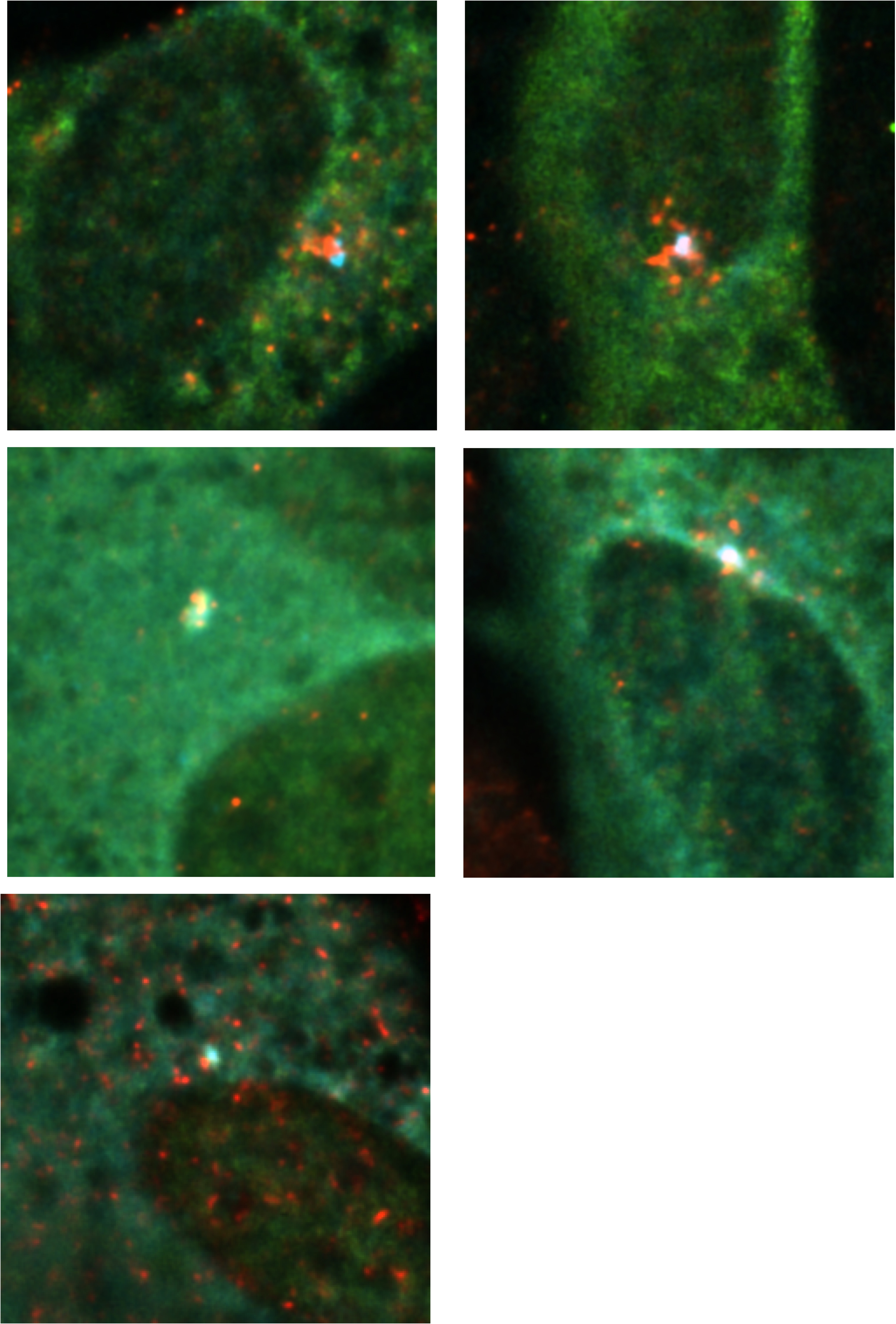

Supplement: Supplementary file 12 — Source data Fig. 5 [file 44318_2025_435_MOESM12_ESM.zip › SD Figure 5/5A.jpg]

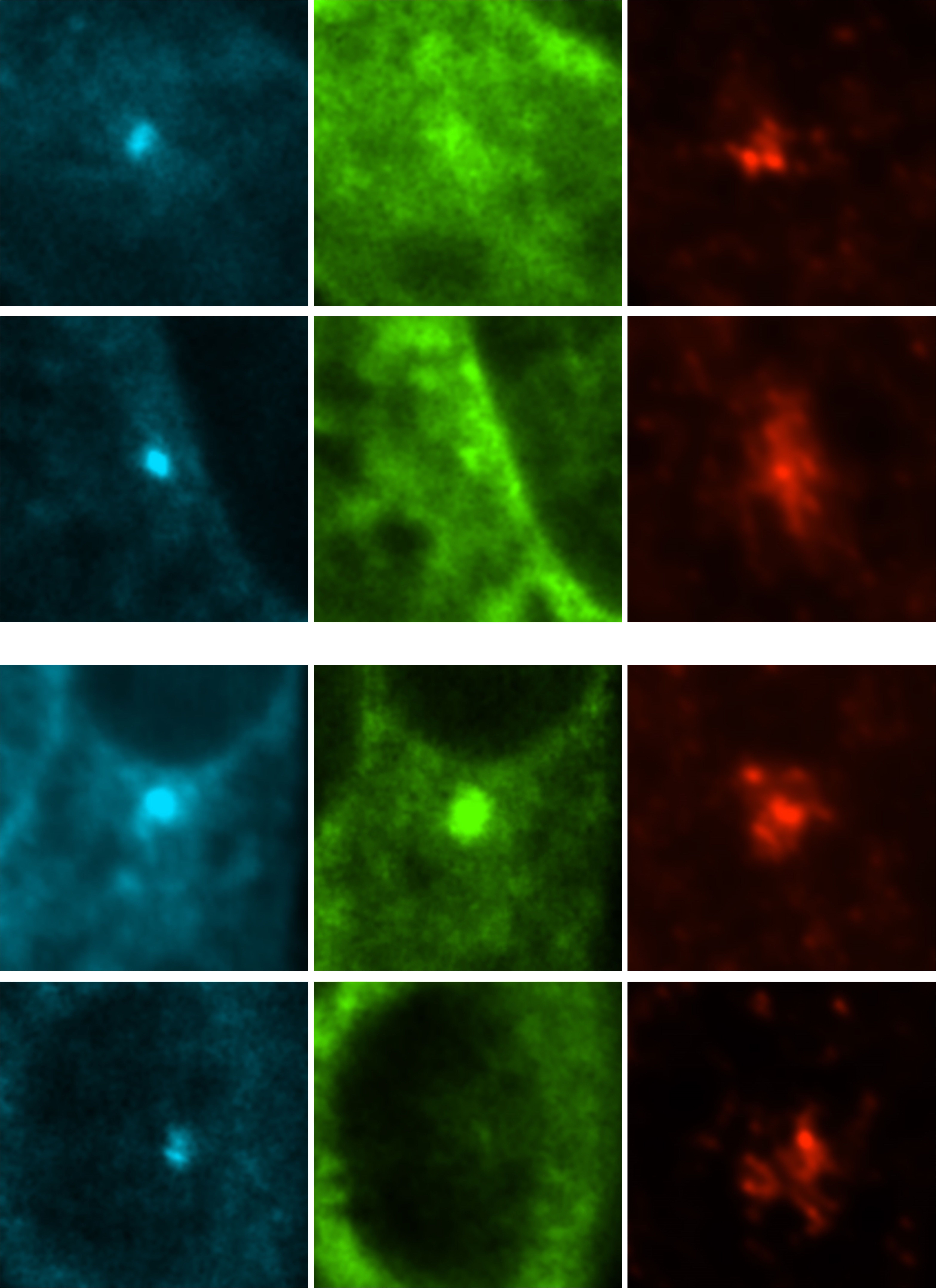

Supplement: Supplementary file 12 — Source data Fig. 5 [file 44318_2025_435_MOESM12_ESM.zip › SD Figure 5/5F.jpg]

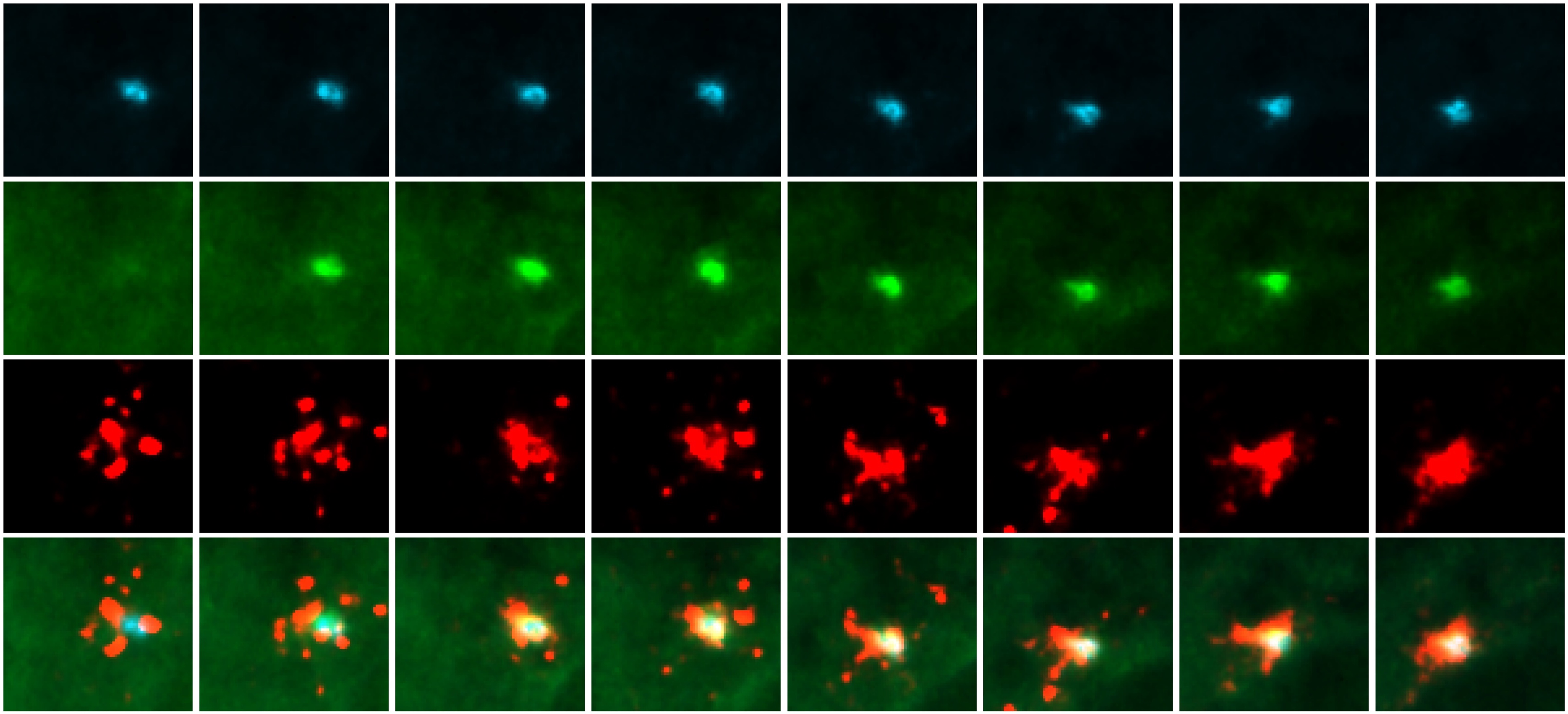

Supplement: Supplementary file 12 — Source data Fig. 5 [file 44318_2025_435_MOESM12_ESM.zip › SD Figure 5/5E.jpg]

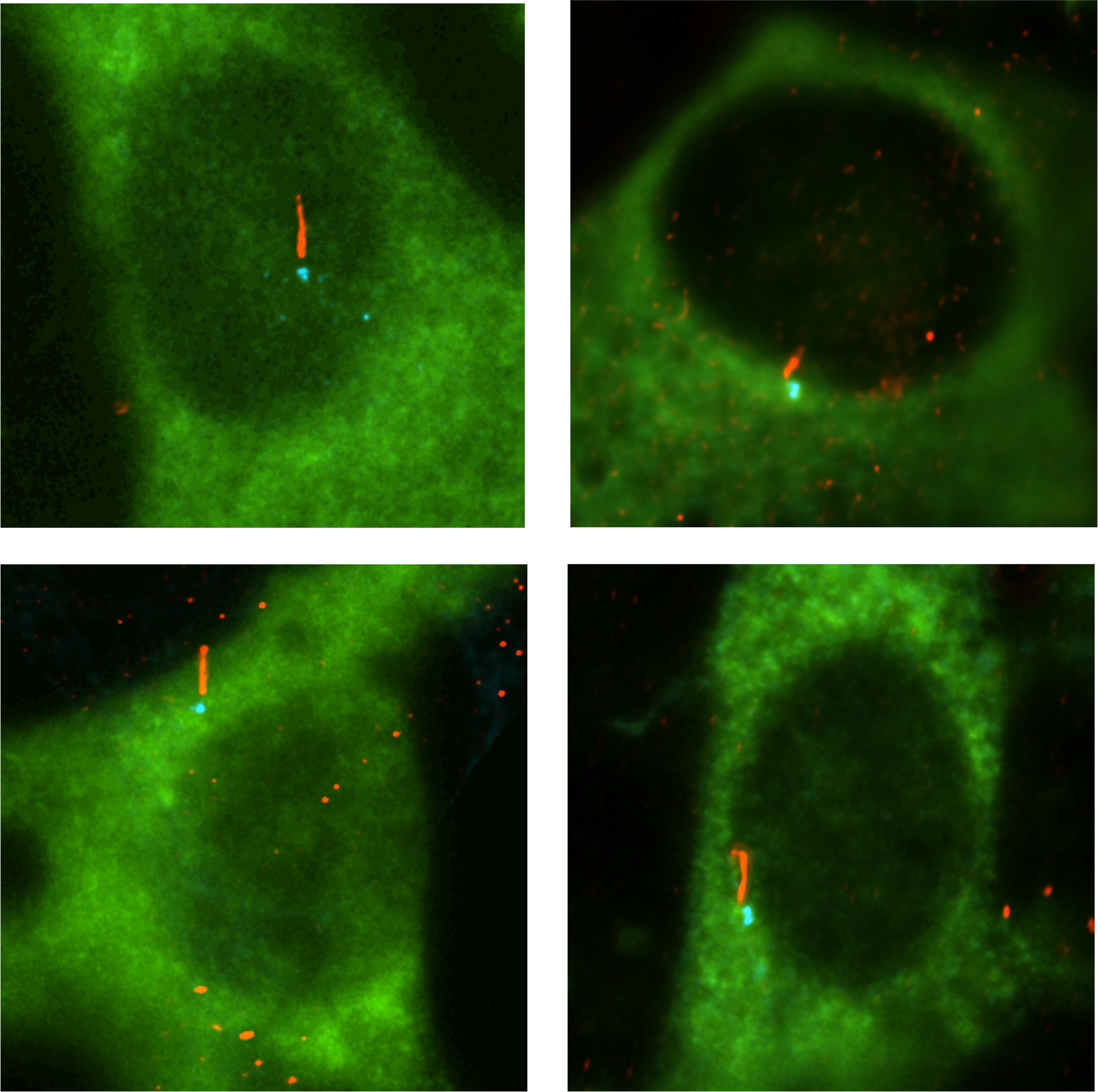

Supplement: Supplementary file 13 — Source data Fig. 6 [file 44318_2025_435_MOESM13_ESM.zip › SD Figure 6/6F.jpg]

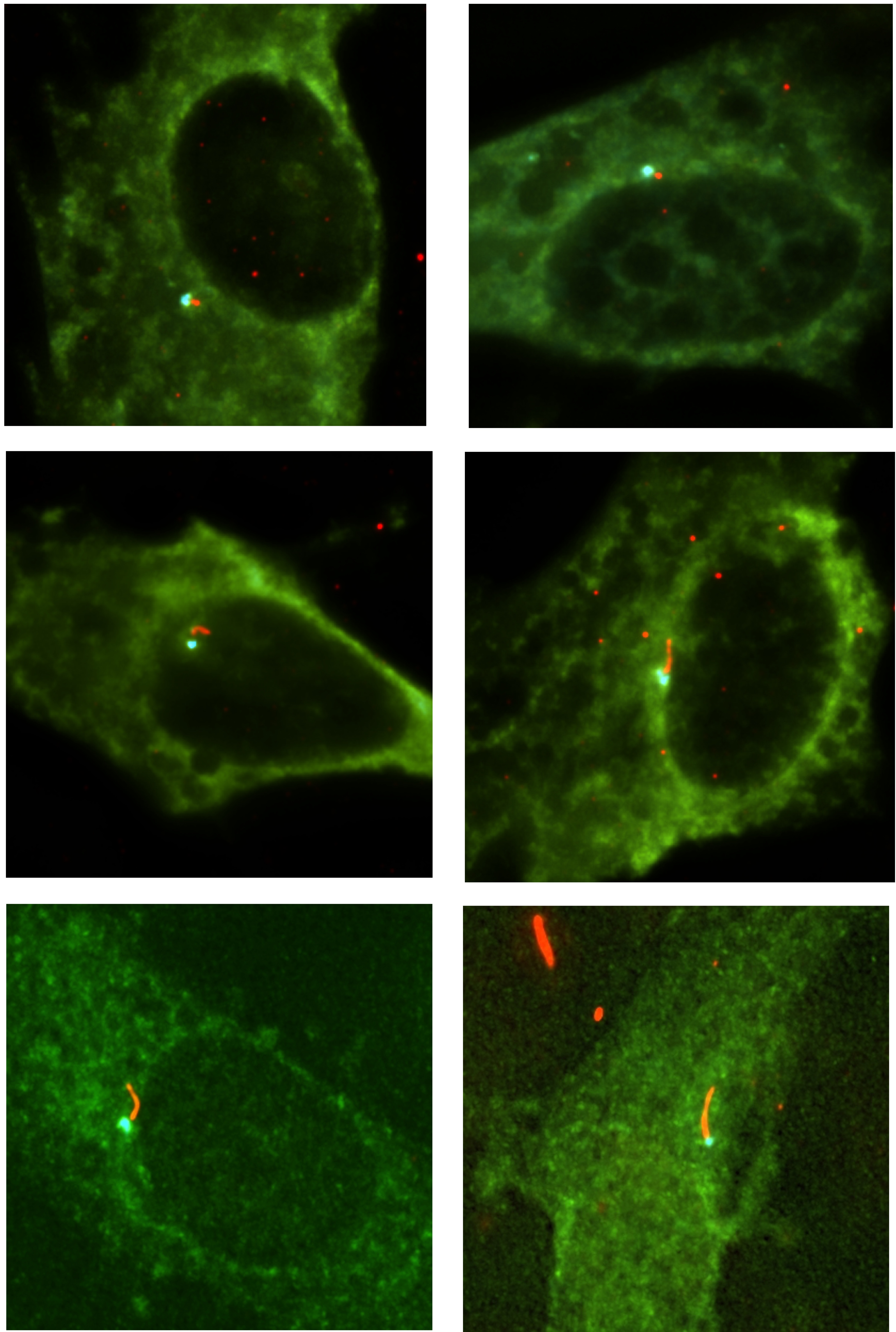

Supplement: Supplementary file 13 — Source data Fig. 6 [file 44318_2025_435_MOESM13_ESM.zip › SD Figure 6/6B.jpg]

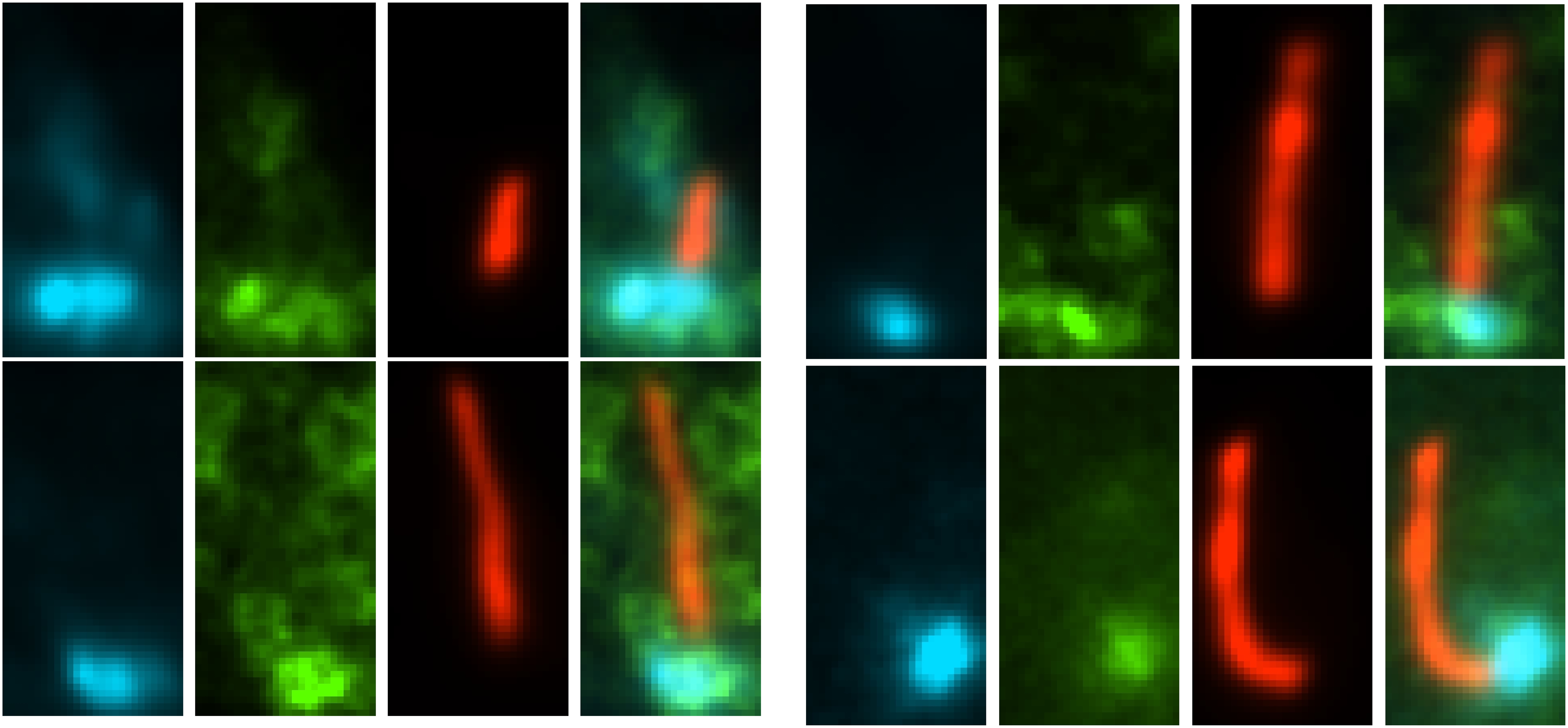

Supplement: Supplementary file 14 — Source data Fig. 7 [file 44318_2025_435_MOESM14_ESM.zip › SD Figure 7/7A.jpg]

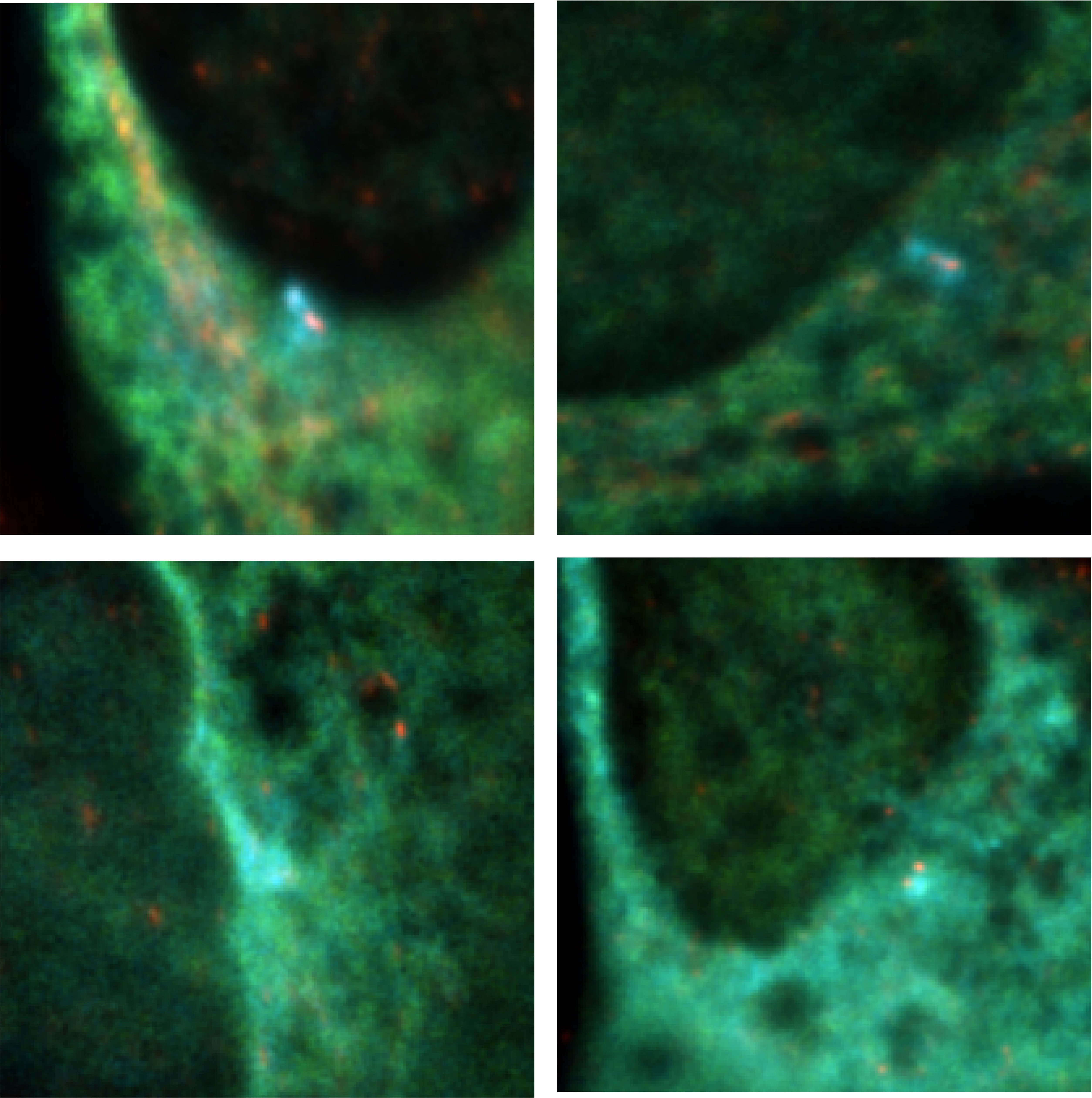

Supplement: Supplementary file 14 — Source data Fig. 7 [file 44318_2025_435_MOESM14_ESM.zip › SD Figure 7/7C.jpg]

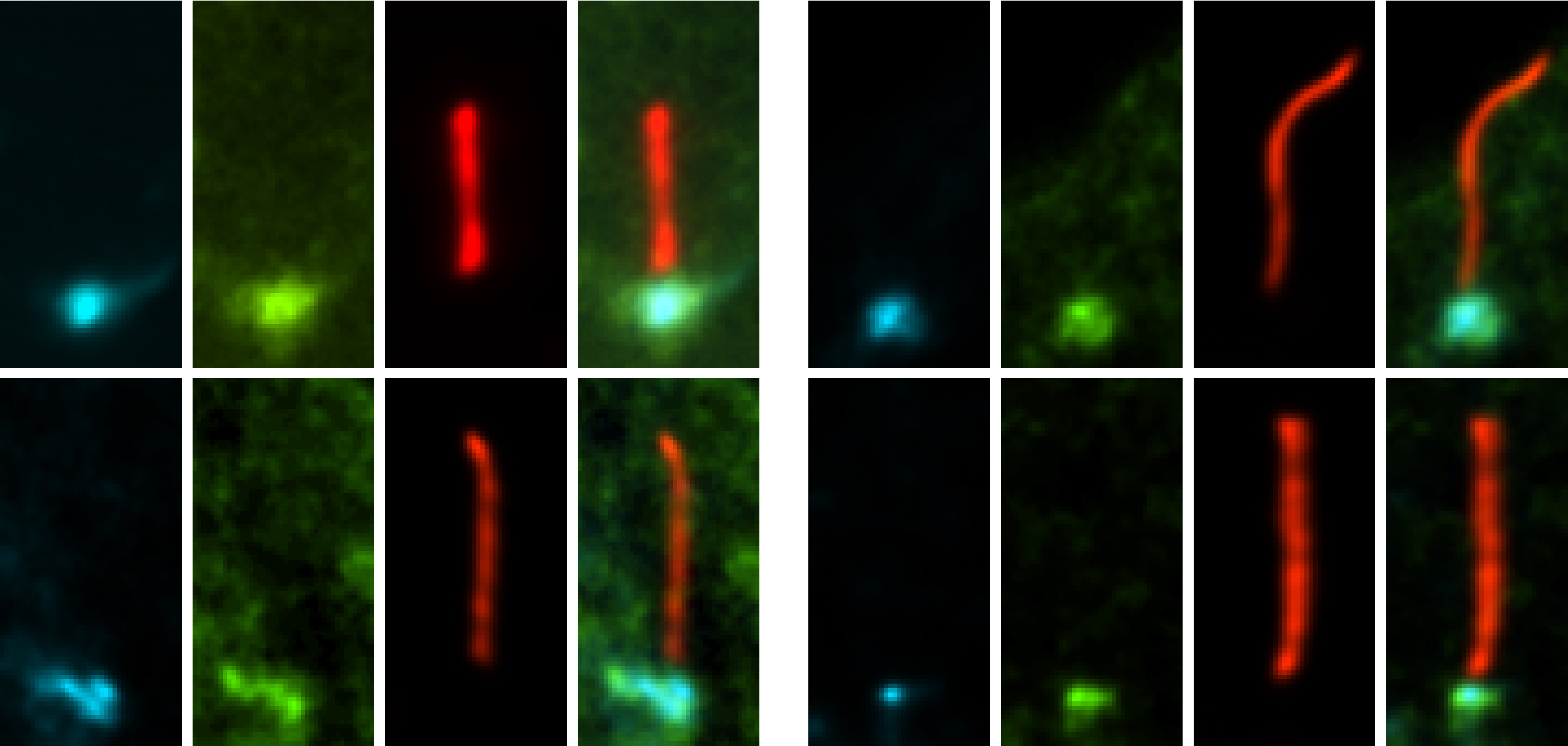

Supplement: Supplementary file 14 — Source data Fig. 7 [file 44318_2025_435_MOESM14_ESM.zip › SD Figure 7/7E.jpg]

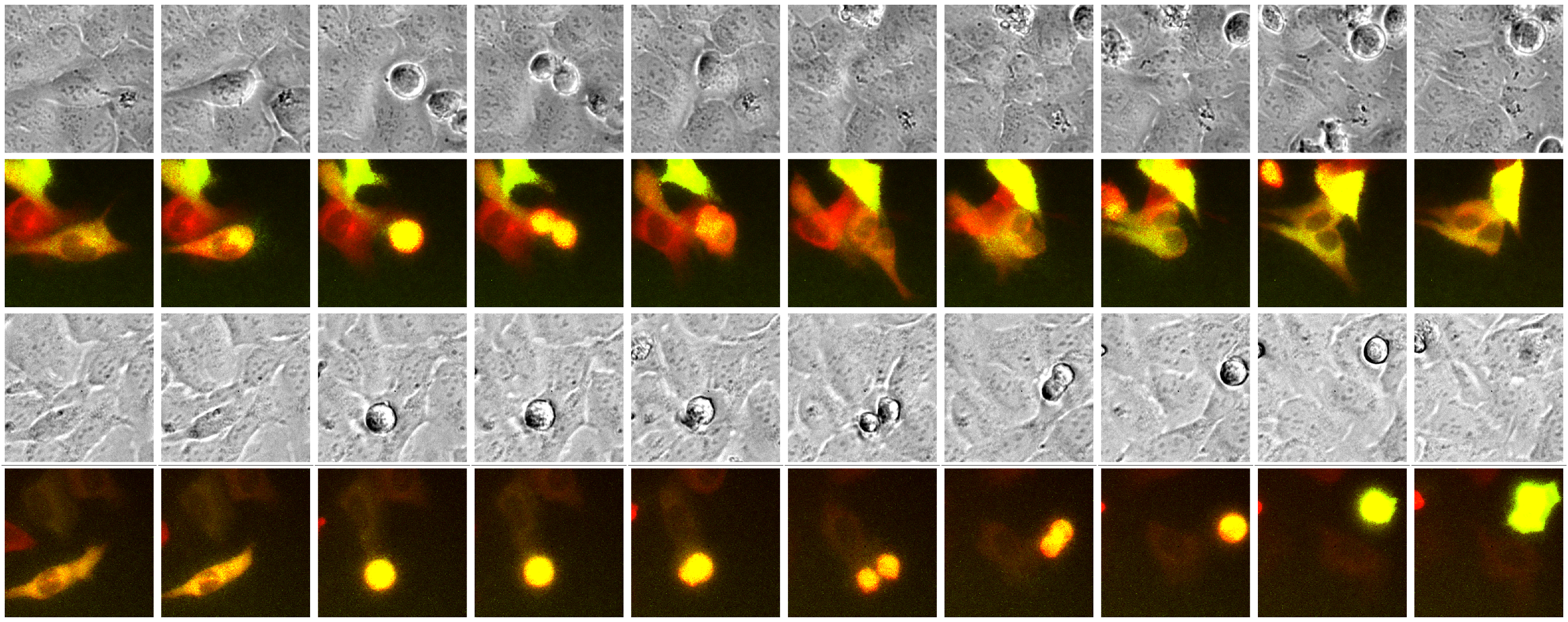

Supplement: Supplementary file 15 — Source data Fig. 8 [file 44318_2025_435_MOESM15_ESM.zip › SD Figure 8/8D.jpg]

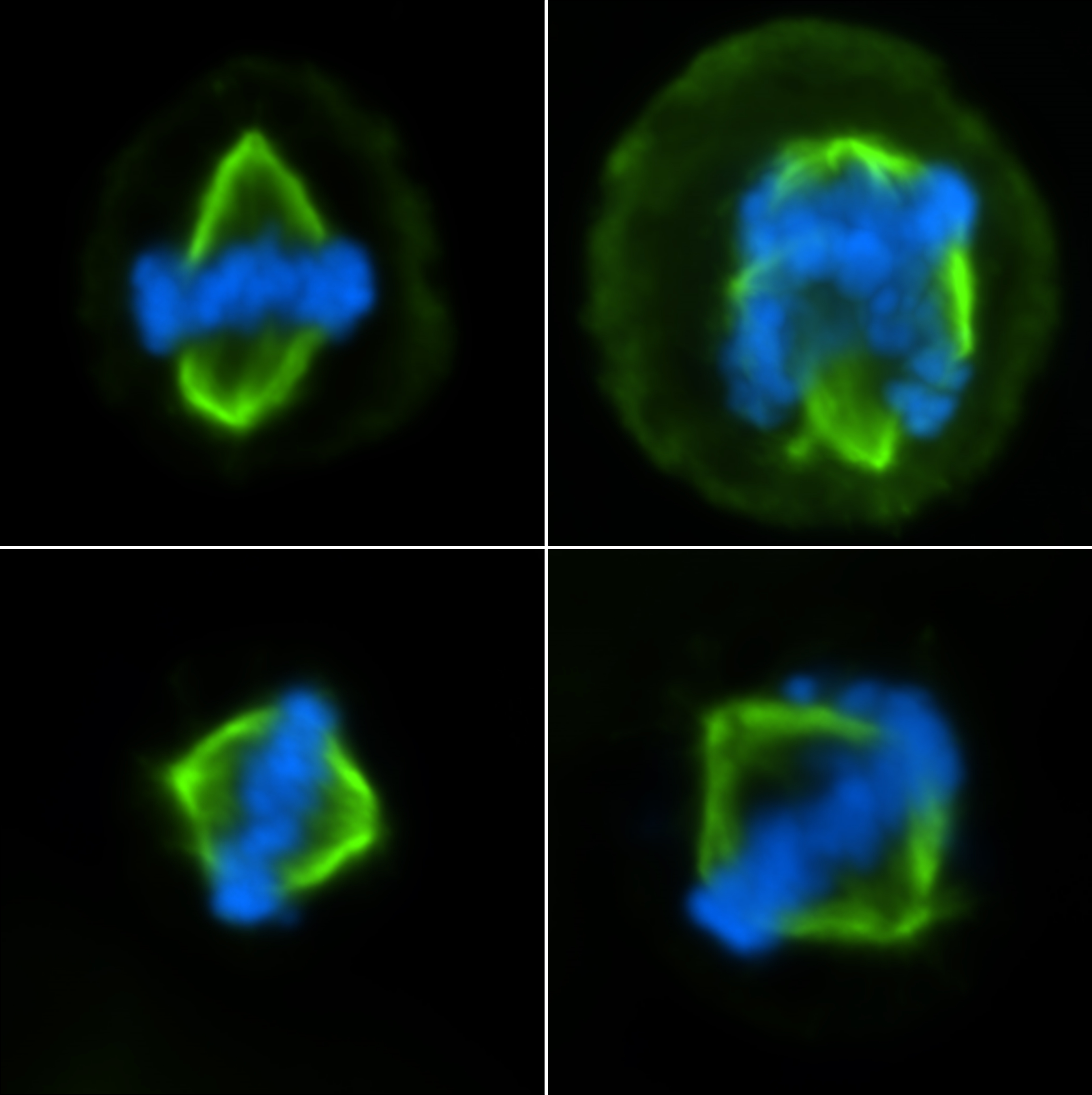

Supplement: Supplementary file 15 — Source data Fig. 8 [file 44318_2025_435_MOESM15_ESM.zip › SD Figure 8/8B.jpg]
