# Supplementary material for: Glutamylation of centrosomes ensures their function by recruiting microtubule nucleation factors
Source: EMBO J. 2025 Apr 14;44(10):2976–96. doi: 10.1038/s44318-025-00435-y (PMC12084555; doi:10.1038/s44318-025-00435-y)
Supplement: Supplementary file 16 — Expanded View Figures [file 44318_2025_435_MOESM16_ESM.pdf]

Expanded View Figures

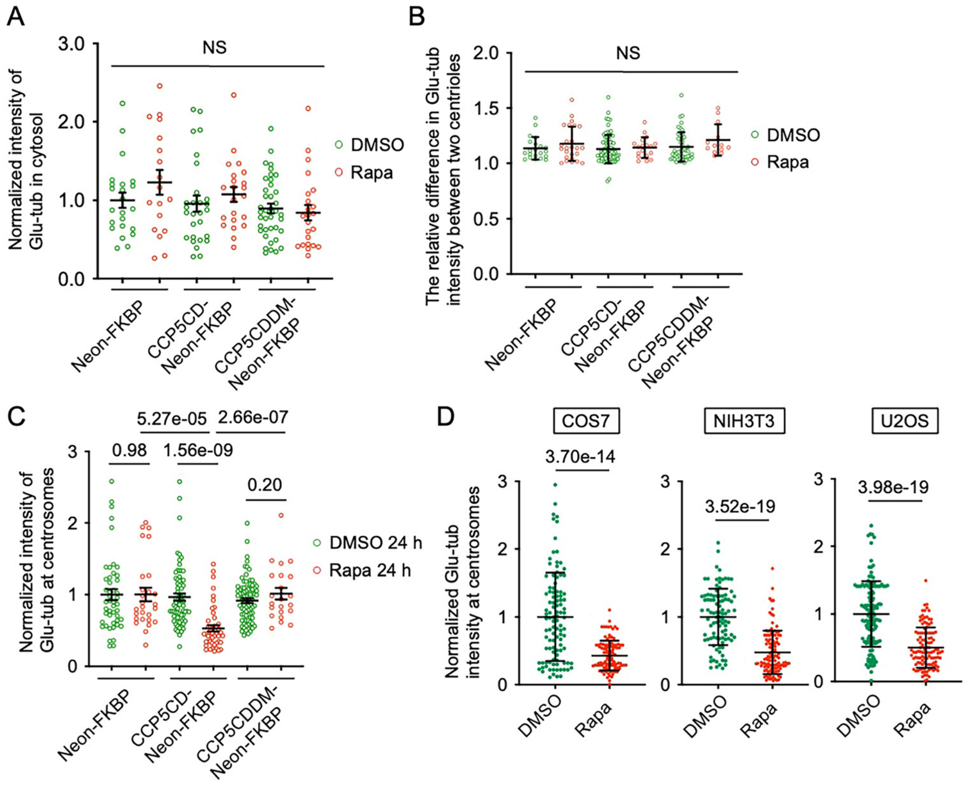

Figure EV1. Specific glutamylation reduction occurs at two centrioles in three different cell types.

(**A–C**) COS7 cells co-transfected with Ce3-FRB-CEP170C and the indicated constructs were treated with either 0.1% DMSO (green circles) or 100 nM rapamycin (Rapa, red circles) for 1 h in (**A**, **B**) or for 24 h in (**C**). Treated cells were immunostained with GT335 antibody to assess the level of glutamylated tubulin in cytosol (**A**); the relative difference in glutamylated tubulin intensity between two centrioles (**B**); and the normalized intensity of centrosomal glutamylation in (**C**). Data are presented as mean ± SEM. *n* (from left to right) = 23, 18, 28, 22, 39, and 23 cells in (**A**); 21, 25, 78, 18, 46, and 13 cells in (**B**); 47, 27, 71, 46, 85, and 22 cells in (**C**), collected from 3 independent experiments. Statistical significance was determined using a one-way ANOVA test. "NS" indicates no significant difference among groups in (**A**, **B**). Student's t tests were performed, and *P* values are indicated in (**C**). (**D**) COS7, NIH3T3, and U2OS cells were transfected with Ce3-FRB-CEP170C and CCP5CD-Neon-FKBP. Transfected cells were treated with 0.1% DMSO (green) or rapamycin (100 nM) for 30 min. The normalized level of tubulin glutamylation at centrosomes under the indicated conditions is shown. Data (black) represent the mean ± SD. *n* = 105, 107, 97, 113, 133, and 112 cells from left to right. Three independent experiments. Student's *t* tests were performed, and *P* values are indicated.

 © The Author(s)

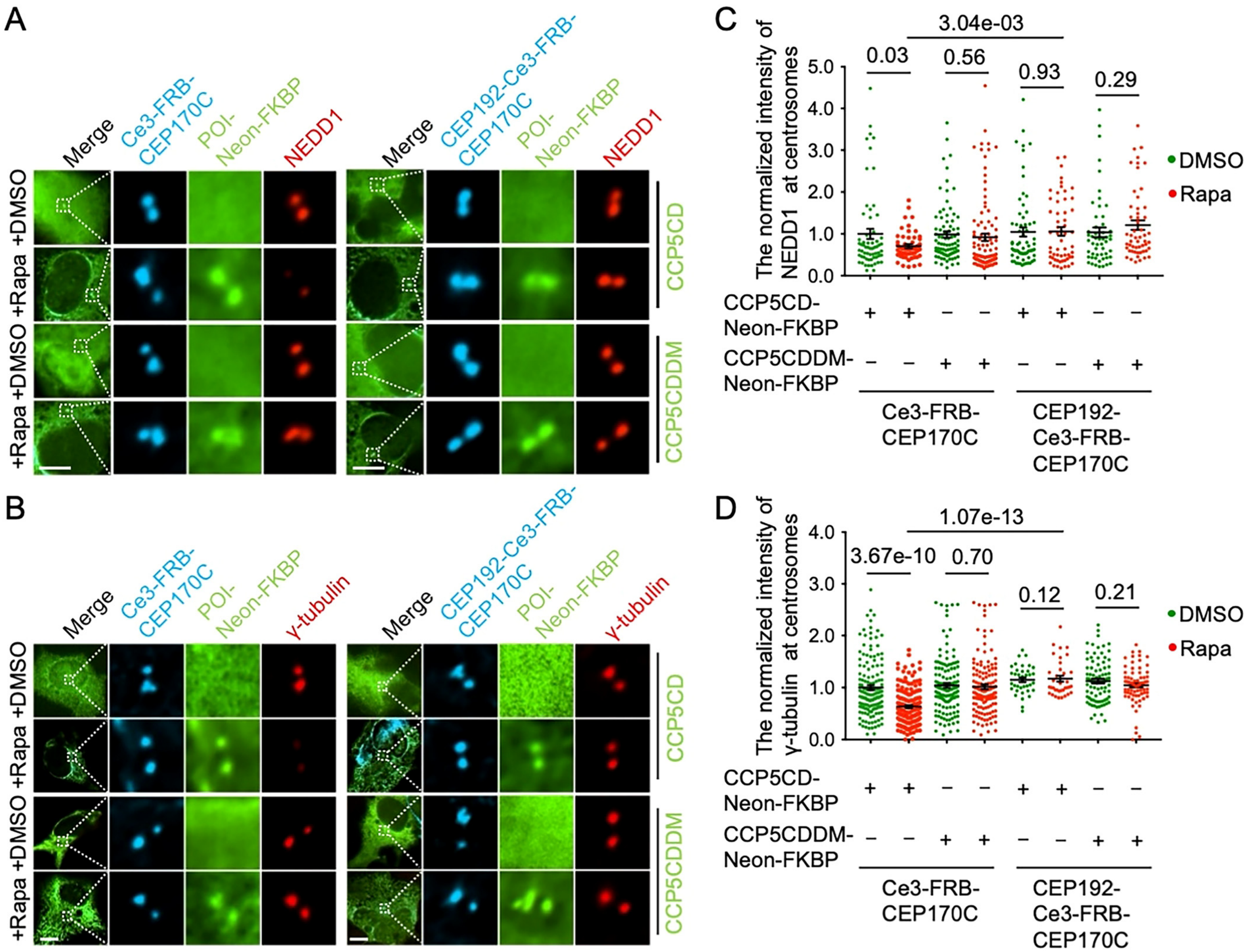

Figure EV2. CEP192 is sufficient to recruit NEDD1 and γ-tubulin to hypoglutamylated centrosomes.

(**A**, **B**) COS7 cells co-transfected with the indicated constructs were treated with DMSO (0.1%) or Rapa (100 nM rapamycin) for 1 h. Following treatment, cells were immunostained with antibodies against NEDD1 (**A**) and γ-tubulin (**B**), respectively. Scale bar, 10 μm. (**C**, **D**) Quantification of the normalized intensity of NEDD1 (**C**) in cells from (**A**) and γ-tubulin (**D**) in cells from (**B**). Data represent as mean ± SEM. *n* (from left to right) = 62, 51, 84, 101, 67, 55, 53, and 54 cells in (**C**); 143, 152, 134, 135, 37, 36, 85, and 69 cells in (**D**); 3–6 independent experiments. Students' t tests were performed, and *P* values are indicated.

© The Author(s)
